# On the structural and practical identifiability of multi‐echo BBB‐ASL tracer kinetic models

**DOI:** 10.1002/mrm.70075

**Published:** 2025-09-10

**Authors:** Tabitha J. Manson, David L. Thomas, Matthias Günther, Lynette J. Tippett, Michael Dragunow, Catherine A. Morgan, Vinod Suresh

**Affiliations:** ^1^ Auckland Bioengineering Institute The University of Auckland Auckland New Zealand; ^2^ School of Psychology and Centre for Brain Research University of Auckland Auckland New Zealand; ^3^ Department of Brain Repair and Rehabilitation, UCL Queen Square Institute of Neurology University College London London UK; ^4^ Imaging Physics Fraunhofer Institute for Digital Medicine MEVIS Bremen Germany; ^5^ MR imaging and spectroscopy, Faculty 1 (Physics/Electrical Engineering) University Bremen Bremen Germany; ^6^ Dementia Prevention Research Clinic University of Auckland Auckland New Zealand; ^7^ Department of Pharmacology and Centre for Brain Research University of Auckland Auckland New Zealand; ^8^ Centre for Advanced MRI, Auckland Uniservices Limited University of Auckland Auckland New Zealand; ^9^ Department of Engineering Science and Biomedical Engineering University of Auckland Auckland New Zealand

**Keywords:** arterial spin labeling, blood–brain barrier, cerebral blood flow, identifiability, water exchange, water permeability

## Abstract

**Purpose:**

Tracer kinetic models are used in arterial spin labeling (ASL); however, deciding which model parameters to fix or fit is not always trivial. The *identifiability* of the resultant system of equations is useful to consider, since it will likely impact parameter uncertainty. Here, we analyze the identifiability of two‐compartment models used in multi‐echo (ME) blood–brain‐barrier (BBB)‐ASL and evaluate the reliability of the fitted water‐transfer rate $(k_{w}$).

**Method:**

The identifiability of two variants of a two‐compartment model (referred to here as “series” and “parallel”) were analyzed using sensitivity matrix and Monte‐Carlo simulation methods, the latter including the effects of noise and fixed‐parameter error. ME‐ASL data were collected at 3T in 25 cognitively normal participants (57–85 y). In one volunteer, additional scans were acquired to estimate noise. Fits for whole‐gray‐matter $k_{w}$ were performed with a theoretically identifiable version of the model.

**Results:**

All models needed one or more fixed parameters to be structurally identifiable, with different combinations required for each. Practical identifiability analysis yielded $k_{w}$ estimates with a median absolute error of 29% (parallel model) and 33% (series model). Fits to data yielded median $k_{w}$ values of 0 (parallel) and 96 min^−1^ (series).

**Conclusion:**

We used identifiability analysis to determine an appropriate BBB‐ASL model for acquired data. Through simulations we showed that parameter estimates depend on model selection and the value of fixed parameters. We demonstrated that fixed‐parameter value and errors significantly impact the reliability of $k_{w}$ values obtained from acquired ME‐ASL images, even with structurally identifiable models.

## 1 INTRODUCTION

The relationship between arterial spin labeling (ASL) signal and physical tissue properties is described using a tracer kinetic model, and single compartment models are commonly used to quantify cerebral blood flow (CBF).^1^ A recent ASL application is the measurement of blood–brain‐barrier (BBB) permeability to water without the use of exogenous contrast agents. Akin to the tracer kinetic models used for DCE MRI, BBB‐ASL uses multi‐compartment models to quantify the rate of exchange of tagged water ($k_{w}$) across the BBB. Typically, these are two‐compartment exchange models (2CXMs) whereby water exchanges from the intravascular to the extravascular extracellular space (IVS‐EES).^2^, ^3^

Estimating model parameters can be challenging when using multi‐compartment models. Usually when fitting for parameter(s) of interest (e.g., $k_{w}$ and CBF), a selection (or all) of the other key parameters (e.g., relaxation time constants) may be fixed. This selection may be made arbitrarily or be informed by the availability of independent measurements. The set of fixed parameters differs between studies, as do the values of the fixed parameters themselves,^4^, ^5^, ^6^, ^7^ reflecting the variation in literature values for the relaxation time constants. Existing estimates for the transverse relaxation time of blood ($T2_{b}$) in particular vary greatly depending on oxygen saturation and hematocrit (HCT),^8^, ^9^ both of which differ in the cerebral capillaries compared to the systemic circulation.^10^, ^11^

A less‐often considered risk in modeling is that of *non‐identifiability*, which occurs if the observed signal is described equally well by multiple sets of model parameter values. Identifiability analysis aims to determine which of a model's free parameters can be uniquely estimated from an experiment's observed output.^12^ Such analyses are broadly categorized into *structural* and *practical* identifiability analyses. A model is said to be structurally identifiable if all its parameters can be uniquely determined when unlimited noise‐free measurements of the model outputs are available. Practical identifiability includes the effect of noise and limited data on parameter identification. To our knowledge, identifiability analyses have not been published for common ASL models.

Here, we analyze the structural identifiability of two commonly‐used BBB‐ASL 2CXMs as well as a single‐compartment perfusion model (1CM) using an approach known as the sensitivity matrix method.^13^ We also use Monte‐Carlo simulation to analyze the structural and practical identifiability of the 2CXMs for a physiological range of parameter values. These results are used to inform the choice of fixed parameters and estimate the accuracy of $k_{w}$ measurements in the context of a multi‐echo (ME)‐ASL experiment. Given the aforementioned variability in values used to date, we also assess the effect of changing the fixed value for $T2_{b}$ on $k_{w}$ estimates acquired from real data.

## 2 METHODS

### 2.1 Theory

#### 2.1.1 Single‐compartment tracer kinetic model

The tracer kinetic model typically used for CBF quantification consists of a single well‐mixed compartment representing the brain tissue in the region of interest (ROI).^14^ Labeled water arrives at the ROI after an arterial transit time (ATT), with the arterial inflow function (AIF) ∆m_a_ describing the time‐course of the bolus inflow. Assuming instantaneous IVS‐EES water exchange and negligible venous outflow, the rate of change of the ASL difference signal (∆M) in the ROI is given by:

(1) $$\frac{d∆M}{d\;t}=\mathrm{CBF}.∆m_{a}-R1_{t}.∆M$$

where $R1_{t}=\frac{1}{T1_{t}}$ is the longitudinal relaxation rate of water in the EES. ∆m_a_ depends on the labeling scheme and for commonly used pseudo‐continuous ASL (pCASL) is given by^14^:

(2) $$\begin{array}{cc} ∆m_{a} & =2.M_{0b}.\alpha .e^{-R1_{b}.\mathrm{ATT}}\;\mathrm{ATT}<t\leq \mathrm{ATT}+\mathrm{BD}\\ & \;=0\;\text{Otherwise} \end{array}$$

where $M_{0b}$ is the longitudinal equilibrium magnetisation of water in blood, α is the labeling efficiency, $R1_{b}=\frac{1}{T1_{b}}$ is the longitudinal relaxation rate of water in blood, and BD is the bolus duration.

#### 2.1.2 Two‐compartment tracer kinetic models

**FIGURE 1:**
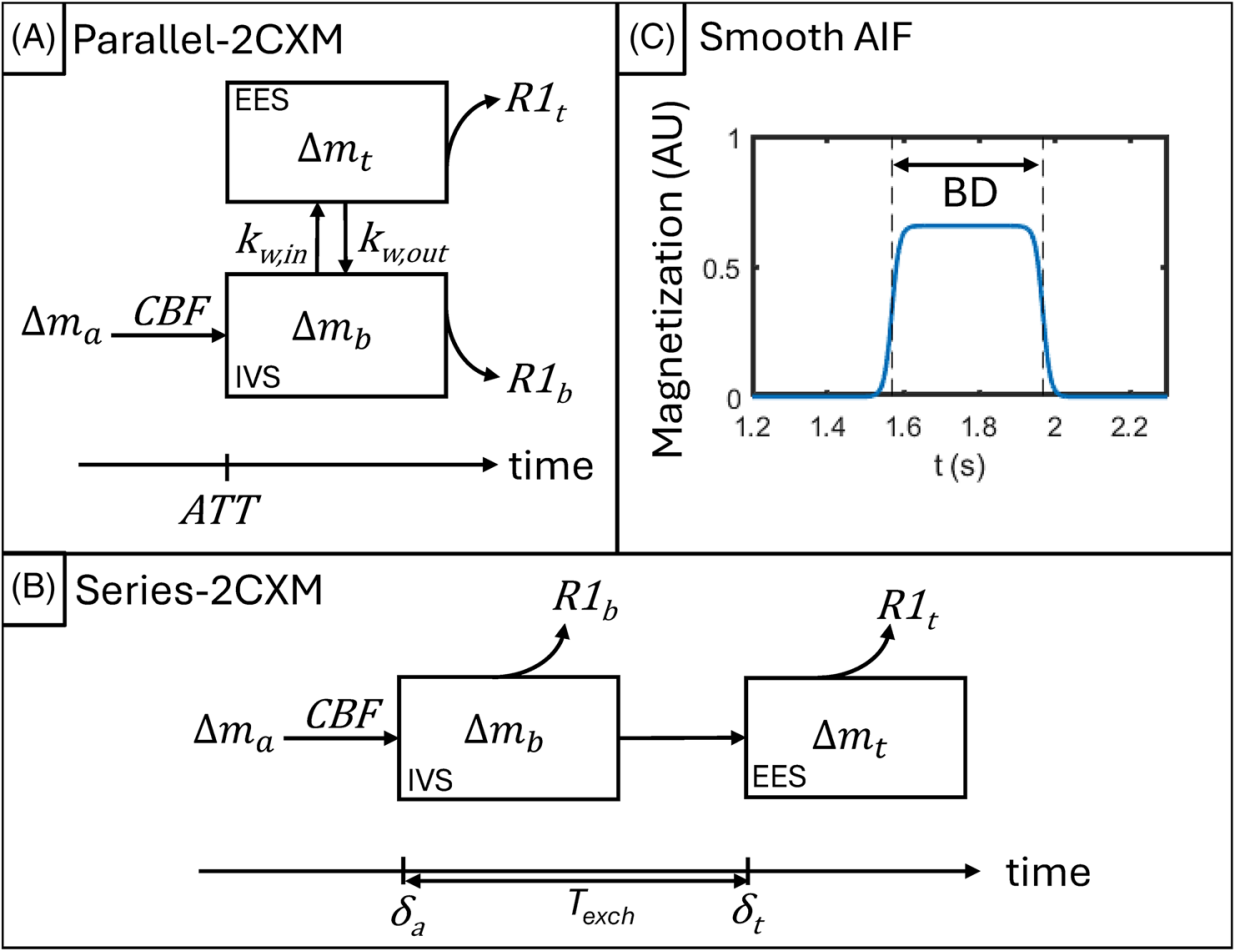
Representation of (A) parallel‐2CXM, and (B) series‐2CXM. Labeled water arrives in the ROI at a rate governed by the CBF and the arterial input function $∆m_{a}$ (described by Eq. 8 and shown in panel C, with longitudinal magnetization plotted against time since the beginning of labeling (t), for $T1_{b}$ =1.65 s, ATT = 1.57 s, BD = 0.4 s). Both models consist of an intravascular space (IVS; containing the blood signal, $∆m_{b}$) and EES (containing the tissue signal, $∆m_{t}$). In the parallel‐2CXM, water exchange is governed by $k_{w,\text{in}}$ and $k_{w,\text{out}}$, with the latter typically assumed to be negligible. In the series‐2CXM, water exchange time $T_{\text{exch}}$ is given by ($\delta_{t}-\delta_{a}$). Signal decay is governed by the longitudinal relaxation rate in each compartment ($R1_{b}$ and $R1_{t}$).

Two commonly used 2CXMs in BBB‐ASL are the St. Lawrence single‐pass‐approximation (SPA) model,^2^ and the Alsop and Detre model,^3^ herein the “parallel” and “series” 2CXMs, respectively. Both models assume two homogeneous compartments (IVS and EES) and negligible venous outflow. The parallel‐2CXM (Figure 1A) is analogous to the two‐compartment Tofts models used in DCE MR imaging,^16^ with the two compartments arranged such that labeled water immediately begins to exchange with the EES at t=ATT. In the series‐2CXM (Figure 1B), labeled water arrives at the ROI at an arterial transit time $\delta_{a}$, after which there is a delay (the exchange time, $T_{\text{exch}}$ ^17^) before the tagged water arrives in the tissue compartment at the tissue arrival time $\delta_{t}.$

For the parallel‐2CXM the equations governing the longitudinal relaxation that occurs prior to the inflow time^18^ (t≤PLD+BD, where PLD is the post‐labeling delay) are:

$$\frac{d\Delta m_{b}}{\mathrm{dt}}=\frac{\mathrm{CBF}}{v_{b}}\Delta m_{a}-\left(k_{w,\text{in}}-k_{w,\text{out}}\right)\;\Delta m_{b}-R1_{b}\Delta m_{b}$$

$$\frac{d\Delta m_{t}}{\mathrm{dt}}=\frac{\left(k_{w,\text{in}}-k_{w,\text{out}}\right)v_{b}}{v_{t}}\Delta m_{b}-R1_{t}\Delta m_{t}\;$$

(3) $$\Delta M_{\text{total}}=v_{b}\Delta m_{b}\left(t\right)+v_{t}\Delta m_{t}\left(t\right)$$

Where $\Delta M_{\text{total}}$ is the observed signal, $\Delta m_{b}$ and $\Delta m_{t}$ are the IVS and EES signal components, the partial volumes of IVS and EES are $v_{b}$ and $v_{t}$, and the water exchange rate $k_{w}$ is given by $\left(k_{w,\text{in}}-k_{w,\text{out}}\right)$ with $k_{w,\text{out}}$ typically assumed to be zero. Eq. 3 can be fitted to single‐echo data,^15^ or ME data with modification; in ME BBB‐ASL, the transverse relaxation rates of each compartment ($R2_{b}$, $R2_{t}$) govern the signal decay during the echo train (after the inflow time). For the parallel‐2CXM, for t>PLD+BD, where the subscript “2” differentiates the post‐inversion signal from the pre‐inversion signal in Eq. 3:

$$\Delta M_{\text{total},2}=v_{b}\Delta m_{b,2}\left(t\right)+v_{t}\Delta m_{t,2}\left(t\right)$$

$$\frac{d\Delta m_{b,2}}{\mathrm{dt}}=-\left(k_{w,\text{in}}-k_{w,\text{out}}\right)\;\Delta m_{b,2}-R2_{b}\Delta m_{b,2}$$

$$\frac{d\Delta m_{t,2}}{\mathrm{dt}}=\frac{\left(k_{w,\text{in}}-k_{w,\text{out}}\right)v_{b}}{v_{t}}\Delta m_{b,2}-R2_{t}\Delta m_{t,2}$$

$$\Delta m_{b,2}\left(t=\mathrm{PLD}+\mathrm{BL}\right)=\Delta m_{b}\left(t=\mathrm{PLD}+\mathrm{BL}\right)\;\text{from}\;\mathrm{Eq}.\;3$$

(4) $$\Delta m_{t,2}\left(t=\mathrm{PLD}+\mathrm{BL}\right)=\Delta m_{t}\left(t=\mathrm{PLD}+\mathrm{BL}\right)\;\text{from}\;\mathrm{Eq}.\;3$$

Alternatively, the signal may be assumed to be governed solely by the transverse relaxation rates of each compartment ($R2_{b}$, $R2_{t}$), neglecting exchange during readout:

$$\frac{d\Delta m_{b,2}}{\mathrm{dt}}\left(t>\mathrm{PLD}+\mathrm{BL}\right)=-R2_{b}\Delta m_{b,2}$$

(5) $$\frac{d\Delta m_{t,2}}{\mathrm{dt}}\left(t>\mathrm{PLD}+\mathrm{BL}\right)=-R2_{t}\Delta m_{t,2}$$

with the same initial conditions as Eq. 4.

The series‐2CXM is given by^19^:

$$∆m_{b}=0\;t<\delta_{a}$$

$$\begin{array}{c} ∆m_{b}=\frac{2M_{0}^{a}.\mathrm{CBF}.\alpha }{v_{b}}\left(\int_{\delta_{a}-t}^{\min\left(\delta_{a}+\mathrm{BL}-t,0\right)}e^{\left(t^{\prime }-\delta_{a}\right)R1_{b}}dt^{\prime }\right)\;\delta_{a}<t\leq \delta_{t}\; \end{array}$$

$$\begin{array}{cc} & =\frac{2M_{0}^{a}.\mathrm{CBF}.\alpha }{v_{b}}\\ & \;\left(\int_{\delta_{a}-t}^{\min\left(\delta_{a}+\mathrm{BL}-t,0\right)}e^{\left(t^{\prime }-\delta_{a}\right)R1_{b}}dt^{\prime }-\int_{\delta_{t}-t}^{\min\left(\delta_{t}+\mathrm{BL}-t,0\right)}e^{\left(t^{\prime }-\delta_{t}\right)R1_{b}}dt^{\prime }\right)\;\delta_{t}<t\; \end{array}$$

$$\begin{array}{c} ∆m_{t}=0\;0<t\leq \delta_{t} \end{array}$$

(6) $$\begin{array}{c} =\frac{2M_{0}^{a}.\mathrm{CBF}.\alpha e^{-\delta_{t}R1_{b}}}{v_{t}}\left(\int_{\delta_{t}-t}^{\min\left(\delta_{t}+\mathrm{BL}-t,0\right)}e^{R1_{t}t^{\prime }}dt^{\prime }\right).\;\delta_{t}<t\; \end{array}$$

where $t^{\prime }$ is a dummy variable. Eq. 6 can be used with single‐echo data. In literature using the series‐2CXM to‐date, the ME signal after the inversion time is given by Eq. 5.^17^, ^20^, ^21^

#### 2.1.3 Identifiability analysis

For a model with parameters p to be structurally identifiable, the observed output y(t,p) must correspond to a single unique point in parameter space. A range of approaches to test this condition exist^22^ and an exemplar using the Laplace transform method^23^ applied to the 1CM is provided in the supplementary material. The structural identifiability analyses that follow are based on the sensitivity matrix approach,^13^ which can be thought of as a hybrid between structural and practical identifiability analysis since the number and spacing of data points (an experimental factor) informs the analysis.

Practical identifiability analysis is an evolving field.^16^ One widely used method is the profile‐likelihood method,^24^, ^25^ used recently to analyze DCE MRI tracer kinetic models.^26^ We have chosen a Monte‐Carlo simulation method,^12^ given its ease of implementation, and the ability to interrogate an expanded parameter space.

##### Structural identifiability analysis by sensitivity matrix

The sensitivity matrix method^13^ is a numerical method that can be used to assess the local structural identifiability of a model under specified experimental conditions. The sensitivity matrix, S, contains the partial derivative of each model output with respect to each system parameter. If S is less than full rank, then the model is not locally structurally identifiable. This occurs when a column of S is null, or when two or more columns of S are co‐linear.

Singular value decomposition (SVD) of S can determine the parameters responsible for non‐identifiability:

(7) $$S=U\sum V^{T}$$

where ∑ contains the singular values of S associated with each parameter. The entries of ∑ can be plotted to visualize the relative sensitivity of each parameter (an identifiability signature) and the elements of $V^{T}$ map the singular values to their corresponding parameter(s).^13^ If a singular value maps to multiple parameters, there are co‐linear columns in S and the model is non‐identifiable. Because of numerical rounding, entries of ∑ and $V^{T}$ will seldom be exactly zero, necessitating practical cutoff values.

Another method to assess co‐linearity is to plot the column vectors of S. When plotted on a log‐scale, the sensitivity curves of co‐linear parameters appear as y‐axis translations.

##### Monte‐Carlo simulation

The sensitivity matrix analysis is limited to assessing local structural identifiability about a single point in parameter space. Monte‐Carlo simulations can be used to determine identifiability of a model over a larger region of parameter space. In the absence of noise or uncertainty in the fixed parameters, a model that is structurally identifiable over the region of parameter space spanned in the simulation should return fitted parameters with minimal error. The practical identifiability of the model can then be tested by introducing noise and fixed‐parameter error.

### 2.2 Data acquisition

The ASL protocol employed in this work was described previously^27^ for $k_{w}$ estimation using a two‐stage approach,^4^, ^28^, ^29^ using a multi‐PLD, single‐echo (SE) sequence for ATT estimation as well as a multi‐PLD, ME sequence for estimating $k_{w}$. Both SE and ME scans used Hadamard‐encoded (HE) pCASL labeling for time‐efficient data‐collection^30^ and were R‐L phase‐encoded. The Hadamard‐decoded PLDs are given Table 1 along with sub‐bolus length and TEs.

**TABLE 1:** Acquisition parameters for the SE and ME scans.

Acquisition parameter	SE scan	ME scan
Sub‐bolus duration (BD) (ms)	400	1000
Decoded PLDs (ms)	100, 500, 900, 1300, 1700, 2100, 2500	100, 1100, 2100
TE (ms)	20.5	20.8, 62.5, 104.2, 145.9, 187.6, 229.2, 270.9
Repeats (n)	1	2
Scan time per repeat	1 min 28 s	2 min 52 s
TR (ms)	3500	
Voxel size (mm^3^)	4 × 4 × 4 (interpolated to 2 × 2 × 4)	

A saturation recovery sequence with 500, 1700, and 2900 ms delay times and two repeats (1 × L‐R, 1 × R‐L phase‐encoding, 35 s each) was used for $M_{0b}$ estimation and distortion correction. A T_1_‐weighted image was also acquired. All scans were performed on a MAGNETOM Skyra 3T MR machine (Siemens Healthcare, Germany) using a 32‐channel head coil.

### 2.3 Identifiability analysis by sensitivity matrix

Identifiability analysis was performed using the sensitivity matrix approach for the SE and ME versions of the 1CM, parallel‐2CXM and series‐2CXM, using the relevant scan parameters. Acquisition parameters are listed in Table 1. System parameters (ϕ) included CBF, $\mathrm{ATT},R1_{b},$ and $R1_{t}$ (all models), $k_{w}$ or $\delta_{t}$ (2CXMs), and $R2_{b}$ and $R2_{t}$ (ME versions of both 2CXMs). Parameter values were chosen according to previous literature estimates for older‐age adults where possible (Table 2).

**TABLE 2:** Physiological parameter values used in numerical identifiability analyses.

Parameter	GT distribution (mean ± SD % where applicable)	Fixed value OR initial value [fitting bounds] in fitting step (mean ±SD%)
ATT (s), $\delta_{a}$ (s)	1.57 ± 15%^35^	Fixed at GT
CBF (mL min^−1^ 100 g^−1^)	48 ± 20%^35^	Fixed at GT
$T1_{b}$ (ms)	1650 ± 5%^36^	Fixed at 1650
$T1_{t}$ (ms)	1330 ± 5%^37^	1330 [±665]
$T2_{b}$ (ms)	110*, ^38^ ±10 %^9^	Fixed at 110 ms
$T2_{t}$ (ms)	70 ± 20%^39^	Fixed at 70 ms
$k_{w}$ (min^−1^)	Uniform distribution, range: 0–500	140^32^ [0, ∞]
$\delta_{t}$ (s)	Non‐normal distribution, mean: 2.00 (see Eq. 11)	2.00 [$\delta_{a,\mathrm{GT}}-\infty$]
v_b_	0.05^40^ (fixed)	Fixed at GT

*Note*: GT values followed literature‐informed normal distributions except for *v*_*b*_ (fixed), $k_{w}$ (uniformly distributed), and $\delta_{t}$ (which was calculated for each instance by Eq. 11 and hence was non‐normal). All physiological parameter values are based on literature estimates for gray matter.

* $T2_{b}$ calculated assuming a cerebral capillary oxygen saturation of 77% and HCT 35% following Zhao et al.^9^

To make the output function continuously differentiable, the AIF (Eq. 2) was replaced with a smooth approximation (Figure 1C):

(8) $$\begin{array}{cc} ∆m_{a} & =2.M_{0b}.\alpha .e^{-R1_{b}.\mathrm{ATT}}\left(\frac{1}{1+e^{-c\left(t-\mathrm{ATT}\right)}}-\frac{1}{1+e^{-c\left(t-\mathrm{ATT}-\mathrm{BL}\right)}}\right)\\ & \;\mathrm{ATT}<t\leq \mathrm{ATT}+\mathrm{BL}\\ & \;=0\;\text{Otherwise} \end{array}$$

with c determining the steepness of the curve. The value c=100 s^−1^ was chosen by visually assessing the impact of c on the 1CM solution curve and ensuring that the rank of S for the 1CM was independent of the specific value of *c* (see supplementary material).

The components of S were calculated for each parameter $\phi_{j}$ as follows:

∆Sij=∂y∂ϕj ti=∂∆Mti,ϕj∂ϕj

(9) $$\approx \frac{∆M\left(t_{i},\phi_{j}+∆\phi_{j}\right)-∆M\left(t_{i},\phi_{j}-∆\phi_{j}\right)}{2\left(∆\phi_{j}\right)}$$

where $∆M\left(t_{i},\phi_{j}\pm ∆\phi_{j}\right)$ was solved using MATLAB's *ode113* solver,^31^ and the size of $∆\phi_{j}$ was the smallest value necessary to achieve the convergence of the entries of S. This was determined by calculating the relative change in each entry, denoted $∆S_{\mathrm{ij}}$, for progressively smaller values of ${∆\phi }_{j}^{q}=1\times 10^{-\left(q-1\right)}\%$ of $\phi_{j}$, q=1,2,… until either all entries of ∆S<10% (the convergence criterion) or until ${∆\phi }_{j}^{q}$ reached the same order of magnitude as computer‐zero (Ɛ) for any parameter without converging:

(10) $$∆S_{\mathrm{ij}}\left({∆\phi }_{j}^{q}\right)=\frac{\left|S_{\mathrm{ij}}({∆\phi }_{j}^{q}\right)\left|-\left|S_{\mathrm{ij}}({∆\phi }_{j}^{q-1}\right)\right|}{\max\left(Ɛ,\;|S_{\mathrm{ij}}\left({∆\phi }_{j}^{q-1}\right)|\right)}$$

Once the convergence criterion was met, ${∆\phi }_{j}^{q-1}$ was used to estimate the entries of S (Eq. 9). After SVD, before calculating the rank of ∑ and before analyzing $V^{T}$, entries of ∑ that were more than 3 decades smaller than the next smallest entry within each respective matrix were treated as negligibly small and rounded down to zero,^13^ as well as any entries of $V^{T}$ that were $<1\times 10^{-3}$.

S was first computed without fixing parameters. For those that were found to be non‐identifiable, the following strategy was used to choose which parameters to fix. First, sets of suspected co‐linear parameters were identified visually using sensitivity plots. Then, one parameter from each pair of co‐linear parameters was fixed. For all models, when in the context of the SE scan, preference was given first to fixing $R1_{b}$, which has a widely used literature value.^1^ For the 2CXMs in the context of the ME scan, preference was given first to fixing parameters that could be independently estimated (i.e., CBF, ATT, and $T2_{t}$ as described in Section 3.3). This procedure was iterated until S was full rank, at which point the model was deemed locally structurally identifiable.

The ME forms of the 2CXMs are discontinuous in time between PLDs, so S was constructed separately for each PLD in the ME scan. Since S would be zero for the 100 ms PLD in this scan (because PLD + BD < simulated ATT), this PLD was omitted from the ME analysis.

### 2.4 Structural and practical identifiability analysis by Monte‐Carlo simulation

Three types of Monte‐Carlo simulations were performed with noise and parameter error added at each iteration, described below. Each simulation had 500 instances, and each instance comprised a data‐generation step and a fitting step. For each instance, a set of noise‐free data points (3x PLDs, 7x TEs per PLD and 2 repeats) was generated. The acquisition parameters (PLD, TE, BD) matched those of the ME scan (Table 1). The physiological parameters were randomly generated from the distributions given in Table 2, and hence were unique for each instance. The forms of the parameter distributions reflected literature values. Since existing literature values for $k_{w}$ are less well established and have a wide range,^4^, ^6^, ^28^, ^32^ ground‐truth (GT) values for $k_{w}$ were generated from a wide uniform distribution, whereas all others were from a normal distribution. For the series‐2CXM, GT values for $k_{w}$ were converted to $T_{\text{exch}}$ and $\delta_{t}$ values by:

$$T_{\text{exch},\mathrm{GT}}=\frac{1}{k_{w,\;\mathrm{GT}}}$$

$$\delta_{t,\mathrm{GT}}=\delta_{a,\mathrm{GT}}+T_{\text{exch},\mathrm{GT}}$$

(11) $$\delta_{a,\mathrm{GT}}={\mathrm{ATT}}_{\mathrm{GT}}$$

Fitting was performed using MATLAB's *lsqnonlin* with a trust‐region‐reflective algorithm.^33^ Simulated data were log‐transformed before fitting to reduce the effect of non‐constant variance on the fit quality.

#### 2.4.1 Simulation 1: Noise‐free simulation without fixed‐parameter errors

Data were generated as described above, without noise. In the fitting step, the decision to fix any given parameter was informed by the sensitivity matrix analysis results. For free parameters, fitting bounds of ±50% of the GT mean value were applied to limit results to physiologically plausible values. Exceptions to this were $k_{w}$ and $\delta_{t}$ which had lower bounds of 0 and $\delta_{a,\mathrm{GT}}$ min^−1^, respectively, and unlimited upper bounds, because of the limited data on plausible values for these parameters. The remaining parameters were fixed at their GT values. Since identifiable parameters are expected to have negligible absolute relative errors (AREs) in this simulation, parameters with median AREs above 1% were deemed non‐identifiable. If $k_{w}$ or $\delta_{t}$ were non‐identifiable, additional parameters were fixed (as per the strategy described in Section 2.2) before running a further simulation.

#### 2.4.2 Simulation 2: Noise‐free simulation with fixed‐parameter errors

Parameters were fixed according to the results from Simulation 1. The data generation and model fitting step was identical to Simulation 1, but with the fixed parameters set to literature values (Table 2) rather than their GT values, thereby introducing error into the fixed parameters.

#### 2.4.3 Simulation 3: Noisy simulation with fixed‐parameter errors

This simulation was the same as Simulation 2, except that a realistic degree of noise was introduced to the simulated signal. An estimate of the noise (variance) of the ME scan was obtained by extending the ME scan from 2 to 10 repeats for a single volunteer—a 64‐y‐old male (not cognitively assessed, but presumed cognitively healthy) who was recruited separately to the other participants (ethical approval by the University of Auckland Human Participants Ethics Committee, ref. 025350). Images were pre‐processed and smoothed as described in Section 2.5. The variance (${\sigma_{i}}^{2}$) of the average gray‐matter signal ∆M at each time point ($t_{i}$, corresponding to PLD + BD + TE) was calculated using each repeat r=1,2,…,10 as follows:

(12) $$\sigma_{i}=\sqrt{\frac{\sum_{r=1}^{R}{\left(∆M_{i,r}-{\bar{\Delta M}}_{i}\right)}^{2}}{R-1}}$$

where

(13) $${\bar{\Delta M}}_{i}=\frac{1}{R}\sum_{r=1}^{R}∆M_{i,r}$$

and R = 10. As the variance was found to be non‐uniform, with higher ΔM values having greater variance, the relationship between ${\sigma_{i}}^{2}$ and ${\bar{\Delta M}}_{i}$ was quantified by fitting a linear model to the log‐transformed ${\sigma_{i}}^{2}$ and ${\bar{\Delta M}}_{i}$ values (using MATLAB's *fitlm* ^34^).

### 2.5 Participants

Participants (6 male, 19 female, mean age 69 years, range 57–85) from an ongoing study by the Dementia Prevention Research Clinic (DPRC),^41^ at The University of Auckland, New Zealand were recruited to undergo an additional scan session, with ethical approval by the University of Auckland Human Participants Ethics Committee (ref. 020737). Participants undertook clinical, medical, and neuropsychological assessments,^41^ and were classified as cognitively normal by a multi‐disciplinary team. Clinical radiology scans were acquired in a separate DPRC scan and assessed by a neuroradiologist to check for exclusionary conditions.

### 2.6 Two‐compartment model fit to real data

**FIGURE 2:**
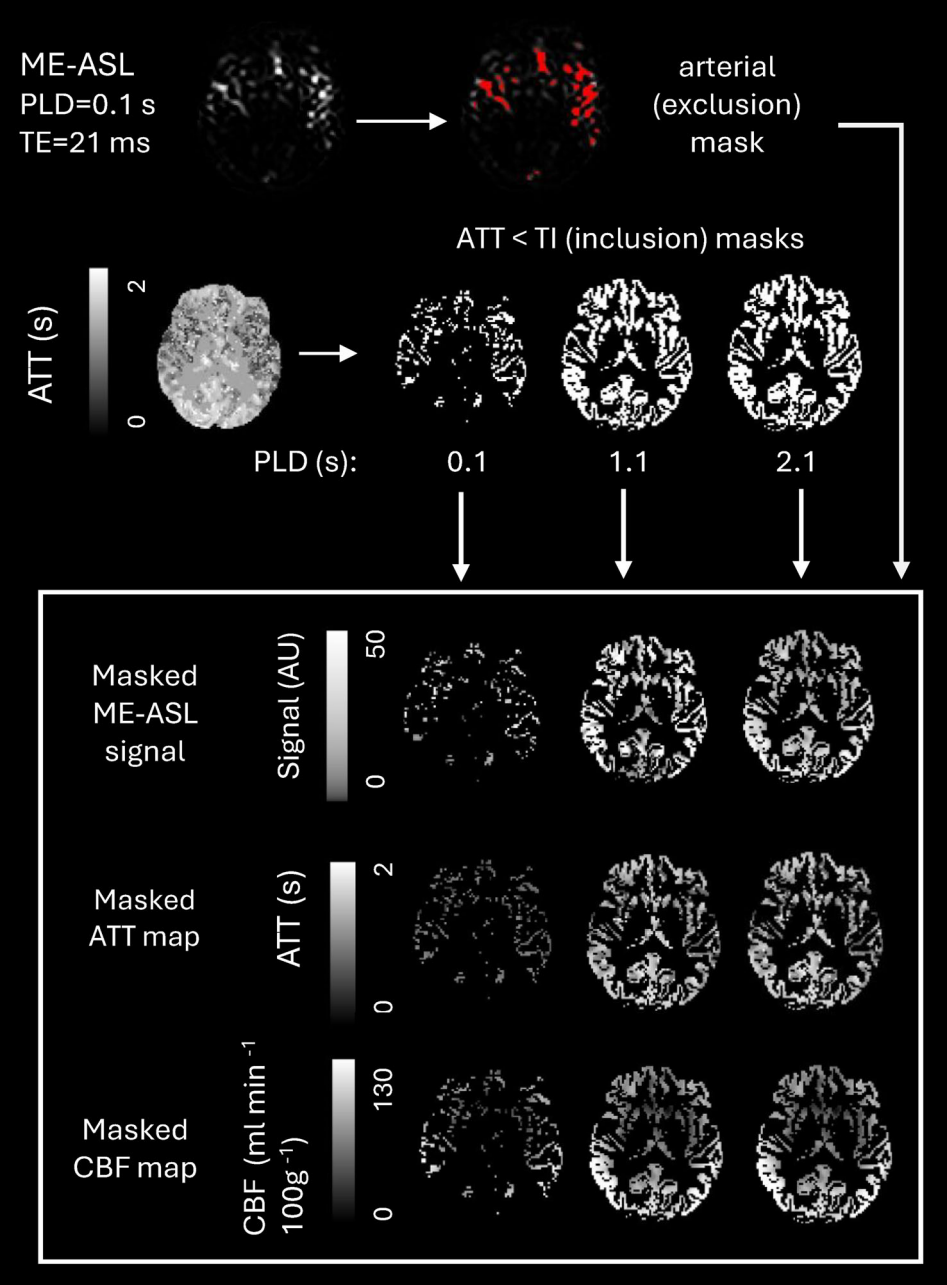
An arterial exclusion mask, created from the 5% of voxels with highest signal intensity in the first PLD and TE of the ME scan was applied prior to the first (ATT and CBF) stage of fitting. Inclusion masks of GM voxels in which ATT <PLD + BD were applied to the GM mask for each participant at each PLD prior to the third ($k_{w}$) fitting stage.

Prior to fitting, images underwent brain‐extraction (*FSL bet* ^42^), motion correction and registration (*FSL mcflirt* ^43^) and distortion correction (*FSL topup* ^44^) followed by Hadamard‐decoding. Gray‐matter segmentation of the T_1_ image was performed using *fsl_anat (v. 6.0.3)*.^45^ A saturation recovery fit for $M_{0b}$ was performed using *asl_calib* ^46^ using white‐matter as the reference tissue. An arterial exclusion mask was made by thresholding the 5% of voxels with the highest signal intensity in the average of the two ME scan PLD = 100 ms, TE = 20.5 ms images (these voxels were excluded from each participant's GM mask, see Figure 2).

Stage 1 (ATT and CBF fitting) was performed using o*xford_asl (v3.9.17)* with partial volume correction^46^, ^47^ and the default 1CM. All data from the SE scan were used to estimate ATT (run 1), and CBF was estimated separately (run 2) using the PLD = 2.1 s, TE = 20.5 ms images from the ME scan (since a single, long PLD image may yield more accurate CBF results than equally‐spaced non‐optimized multi‐PLD data^48^). ATT was initialized at 1.3 s for both runs, and fixed at this value for run 2.

Stage 2 ($T2_{t}$ fitting) involved fitting for {A,R2t,c} in the following mono‐exponential model:

(14) $$M_{\text{summed}}\left(\mathrm{TE}\right)=A.e^{-\mathrm{TE}.R2_{t}}+c$$

where $M_{\text{summed}}$ is the sum of all HE data (equal‐parts labeled/un‐labeled) at each TE.

Stage 3 ($k_{w}$ fit) involved fitting both 2CXMs to all ME scan images, which were smoothed using a 2D 8 mm FWHM Gaussian kernel. The ATT map was used to create ATT <(PLD + BD) binary masks for each PLD (Figure 2), which were multiplied with the GM masks prior to taking the $\Delta M_{\text{total}}$ GM‐averages at each PLD and TE and fitting the log of Eqs. 5 and 6. The ATT and CBF maps were masked in the same way—so that the GM‐average ATT and CBF values only included voxels in which labeled bolus was actually present at each PLD.

Additionally, to assess the effect of changing the fixed value of $T2_{b}$ on $k_{w}$, the fit was repeated using two alternative fixed values for $T2_{b}$ (80 ms and 165 ms, corresponding to O_2_sat of 0.7 and 0.9, respectively, capillary HCT = 35%^38^).

## 3 RESULTS

### 3.1 Identifiability analysis by sensitivity matrix

**FIGURE 3:**
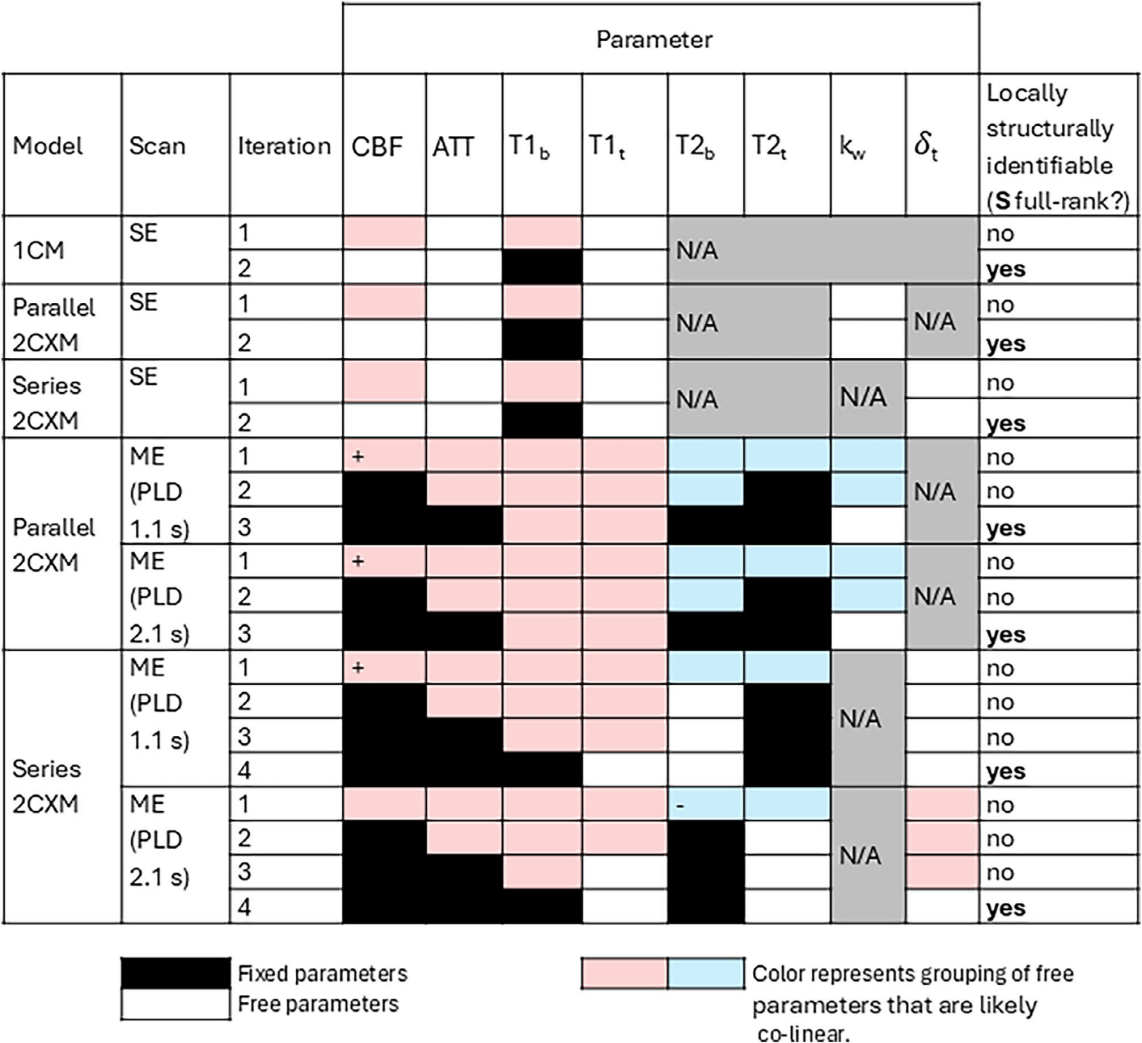
Summary of numerical structural identifiability results at one physiologically plausible point in parameter space. All cells shaded the same color represent parameters that appeared co‐linear from inspecting the sensitivity plots for that iteration. A +/− in a cell denotes high/low sensitivity apparent from the sensitivity plots. Combinations with a full‐rank sensitivity matrix S are locally structurally identifiable.

The numerical structural identifiability result for every combination of fixed parameters that was assessed is presented in Figure 3. All models were assessed first in conjunction with the SE scan, and both 2CXMs were then assessed in conjunction with the ME scan.

#### 3.1.1 SE scan

**FIGURE 4:**
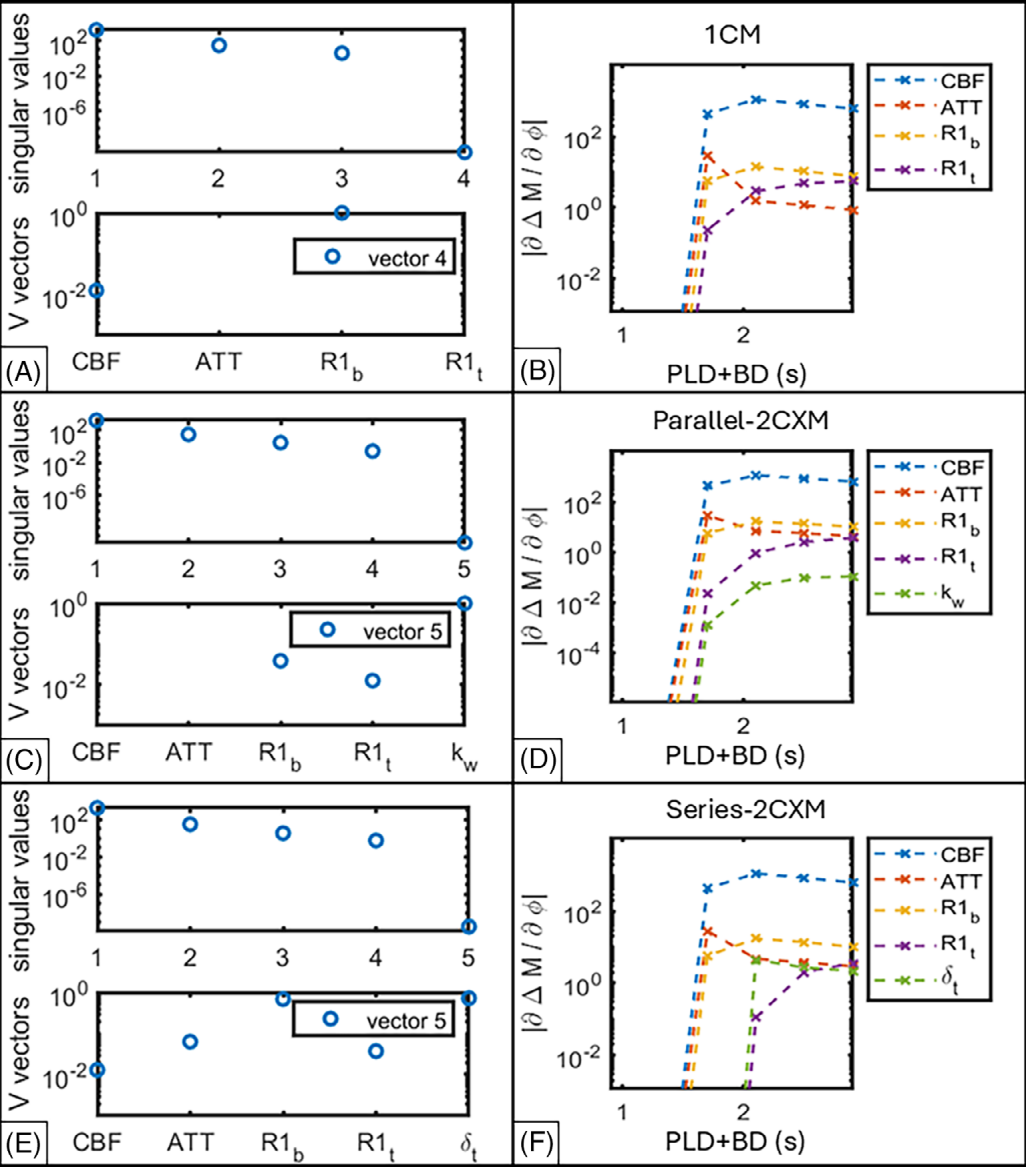
Identifiability signatures for the SE scan (A, single‐compartment model; C, parallel‐2CXM; E, series‐2CXM). In panels A, C, and E, the top plot shows the singular values of S in descending order, with a gap of more than three decades between singular values indicating non‐identifiability; the bottom plot shows the absolute values of the column vectors of $V^{T}$ relating the non‐identifiable parameters to the small singular value(s) of S. Only $\left|V^{T}\right|$ values >0.001 are shown. Sensitivity plots are shown on the right for each model (B, single‐compartment model; D, parallel‐2CXM; F, series‐2CXM.

Figure 4 shows the identifiability signatures and sensitivity plots for all three models when applied to the SE scan, starting with the 1CM. The top plot in Figure 4A shows a gap exceeding three decades between the third and fourth singular values of S, so rank(S)=3 (less than full rank), indicating non‐identifiability.

The bottom plot of Figure 4A shows the singular vector of V pertaining to the last singular value (i.e., the last column vector of $V^{T}$) for the 1CM. There are two parameters ($\mathrm{CBF},R1_{b})$ with non‐zero contributions to the fourth vector, indicating co‐linearity between these parameters (in agreement with the analytical structural identifiability analysis that was performed for this model by Laplace transform—see supplementary material). The sensitivity plot (Figure 4B) supports this, with the curves for CBF and $R1_{b}$ appearing as y‐axis translations of each other. Fixing $R1_{b}$ resulted in the 1CM being locally structurally identifiable (Figure 3, result row 2).

Both 2CXMs were also structurally non‐identifiable with the SE scan, with gaps between the fourth and fifth singular values exceeding three decades (Figure 4C,E, top plots). All parameters for the series‐2CXM, and $R1_{b},R1_{t}$ and $k_{w}$ for the parallel‐2CXM were locally structurally non‐identifiable (Figure 4C and E, bottom panels). The sensitivity plots for both models (Figure 4D,F) suggest co‐linearity between CBF and $R1_{b}$. Fixing $R1_{b}$ resulted in both 2CXMs being locally structurally identifiable with the SE scan.

CBF was a high sensitivity parameter for all models (evidenced by high values of $\left|\frac{\partial ∆M}{\partial \phi }\right|$ in Figure 4B,D,a,n,D,F) whereas water exchange was a low sensitivity parameter for both 2CXMs (lower values of $\left|\frac{\partial ∆M}{\partial \phi }\right|$ in Figures D and F), especially the parallel‐2CXM.

#### 3.1.2 ME scan

**FIGURE 5:**
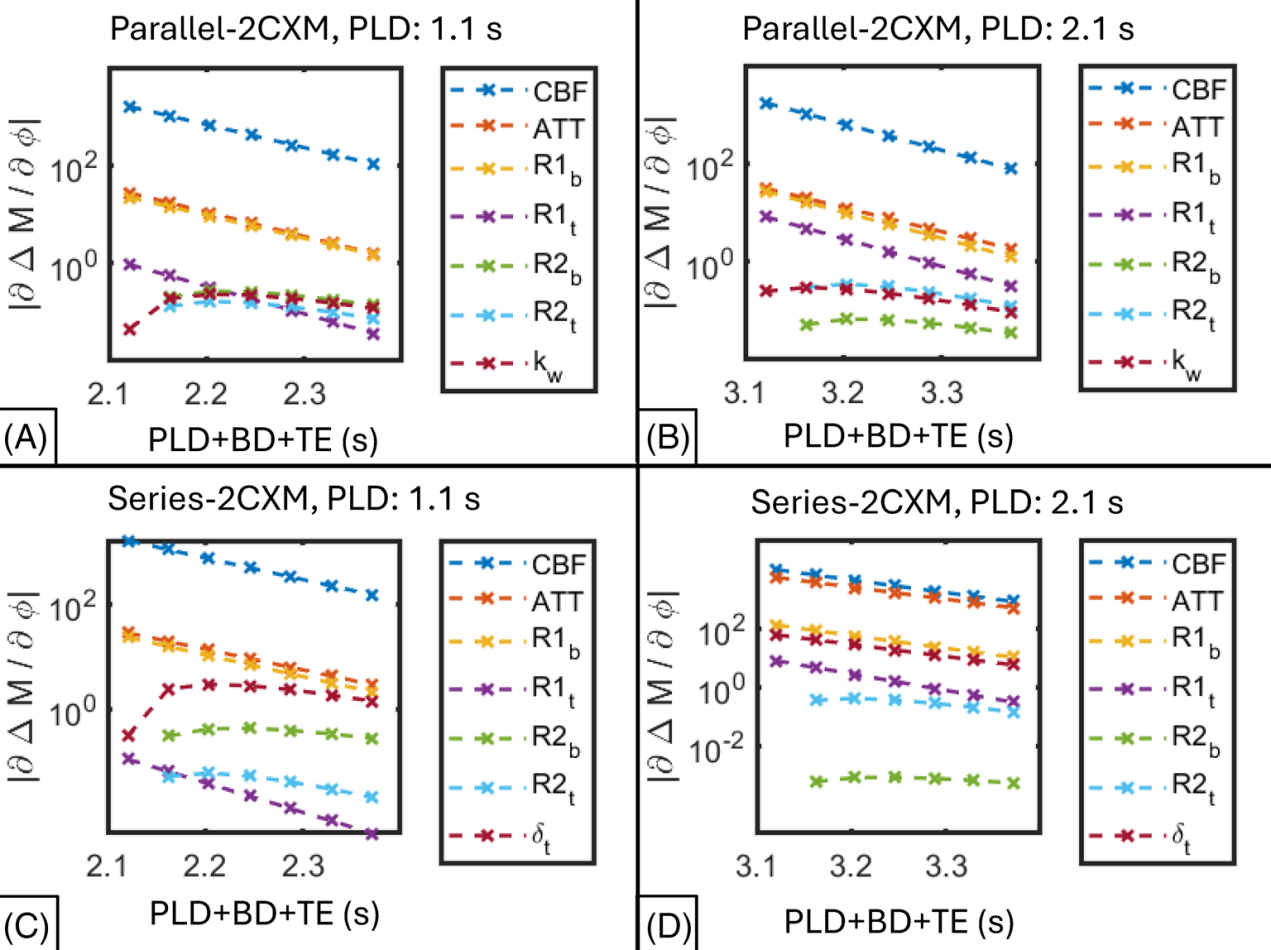
Sensitivity plots for the 2CXMs when used in conjunction with the ME scan. The acquisition time, PLD + BD + TE, is given in seconds. (A) Parallel‐2CXM at PLD = 1.1. (B) Parallel‐2CXM at PLD = 2.1 s. (C) Series‐2CXM at PLD = 1.1 s. (D) Series‐2CXM at PLD = 2.1 s.

When used with the ME scan, both 2CXMs had multiple co‐linear parameters, making the identifiability signatures harder to interpret; therefore, only the sensitivity plots are presented in Figure 5. For completeness, identifiability signatures are provided as supplementary material (Figures S1 and S2).

The parallel‐2CXM sensitivity plots (Figure 5A,B) show that this model's output is much more sensitive to CBF than the other parameters, and the sets $\left\{\mathrm{CBF},\mathrm{ATT},R1_{b},R1_{t}\right\}$ and $\left\{R2_{b},R2_{t},k_{w}\right\}$ appeared possibly co‐linear at both PLDs. Fixing all but $R1_{b},R1_{t},$ and $k_{w}$ resulted in S being full rank (Figure 3), meeting our criteria for local structural identifiability. It is noteworthy that $\left\{R1_{b},R1_{t}\right\}$ appeared co‐linear in the sensitivity plots for this iteration, as this informed the subsequent fixing of $R1_{b}$—see section 3.2.1.

The series‐2CXM sensitivity plots (Figure 5C,D) show that the output is much more sensitive to CBF than other parameters at PLD = 1.1 s, and much less sensitive to $R2_{b}$ than other parameters at PLD = 2.1 s. The sets $\left\{\mathrm{CBF},\mathrm{ATT},R1_{b},R1_{t}\right\}$ and $\left\{R2_{b},R2_{t}\right\}$ appeared co‐linear at PLD = 1.1 s, and $\left\{\mathrm{CBF},\mathrm{ATT},R1_{b},R1_{t},\delta_{t}\right\}$ and $\left\{R2_{b},R2_{t}\right\}$ appeared co‐linear at PLD = 2.1 s. Fixing all but $R1_{t}$, $R2_{b}$ and $\delta_{t}$ resulted in local structural identifiability at PLD = 1.1 s, and fixing all but $R1_{t}$, $R2_{t}$ and $\delta_{t}$ yielded local structural identifiability at PLD = 2.1 s (Figure 3, result rows 16 and 20).

### 3.2 Structural and practical identifiability analysis by Monte‐Carlo simulation (ME scan)

Monte‐Carlo simulations allowed the performance of models that were found to be locally structurally identifiable at the chosen literature values to be assessed over a wider region of parameter space. For brevity, we test this only for the 2CXMs in conjunction with the ME scan. We assume CBF and ATT can be measured independently with negligible error. We also assume that $T2_{t}$ can be identified with negligible error by fitting a mono‐exponential curve to the static ME data.

ARE values are provided as medians rather than means for each simulation to minimize the effect of outliers. All simulation results are presented in Table 3.

**TABLE 3:** Monte‐Carlo simulation results.

Simulation 1: Noise‐free simulation without fixed parameter error							
Parameter median absolute relative error (%)							
Model	Iteration	$T1_{b}$ (s)	$T1_{t}$ (s)	$T2_{b}$ (s)	$k_{w}$ (min^−1^)	$\delta_{t}$ (s)	$T_{\text{exch}}^{-1}$ (min^−1^)
Parallel 2CXM	1	1.48	5.57	–	9.81	N/A	N/A
	2	–	0.00	–	0.00		
Series 2CXM	1	–	3.66	5.00	N/A	7.40	37.84
	2	–	0.33	–		0.06	0.38

Simulation 2: Noise‐free simulation with fixed parameter error							
Parameter median absolute relative error (%)							
Parallel 2CXM	1	3.76	8.28	5.98	28.7	N/A	N/A
	2*	3.56	3.58	6.39	36.3		
Series 2CXM	1	3.38	8.5	6.61	N/A	4.85	32.60
	2*	3.38	10.22	6.66		4.9	32.13

Simulation 3: Noisy simulation with fixed parameter error							
Model		$T1_{b}$ (s)	$T1_{t}$ (s)	$T2_{b}$ (s)	$k_{w}$ (min^−1^)	$\delta_{t}$ (s)	$T_{\text{exch}}^{-1}$ (min^−1^)
Parallel 2CXM	Mean (SD)	1.65	1.38 (0.35)	0.11	925.7 (761.8)	N/A	N/A
	Median (LQ, UQ)	1.65	1.33 (1.19, 1.48)	0.11	252.8 (127.2, 423.6)		
	Median ARE (%)	3.56	10.4	6.39	32.13		
Series 2CXM	Mean (SD)	1.65	1.43 (0.42)	0.11	N/A	1.99 (0.68)	3.63 × 10^10^ (7.90 × 10^11^)
	Median (LQ, UQ)	1.65	1.32 (1.19, 1.50)	0.11		1.90 (1.67, 2.12)	224.7 (112.6, 434.19)
	Median ARE (%)	3.38	10.2	6.66		4.9	33.1

*Note*: Simulation 1: 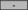 Parameter fixed at GT value (ARE = 0). Simulation 2 and 3: 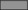 Parameter fixed at literature value. Simulations were iterated, fixing different parameters with each iteration based on the previous result. Results for simulations 1 and 2 show the ARE in each parameter for each iteration (parameters fixed at literature or GT values are grayed‐out). Simulation 2, iteration 2* was performed after the first fit to real data, to check the $k_{w}$ ARE when all other parameters were fixed—see Section 3.3. For the final simulation (simulation 3), median and mean values for each parameter are given in addition to the ARE.

#### 3.2.1 Simulation 1: Noise‐free simulation without fixed‐parameter error

For the parallel‐2CXM, initially $T1_{b}$, $T1_{t}$ and $k_{w}$ were left free, and the remaining parameters were fixed (informed by the sensitivity matrix results—see Figure 3, rows 9 and 12). The simulation resulted in the ARE for $k_{w}$ being 9.8%, which is structurally non‐identifiable by our criteria of ARE >1%, however fixing $T1_{b}$ at its GT value in a subsequent simulation yielded error‐free estimates of both remaining free parameters ($T1_{t}$ and $k_{w}$). The series‐2CXM was also non‐identifiable in the first simulation ($\delta_{t}$ ARE 7.4%). Fixing $T2_{b}$ at its GT value in a subsequent simulation brought the ARE for the remaining free parameters ($\delta_{t}$ and $T1_{t}$) under 1%.

#### 3.2.2 Simulation 2: Noise‐free simulation with fixed‐parameter error

This simulation was performed for the locally structurally identifiable versions of each model identified in Simulation 1 (i.e., $T1_{t}$ and $k_{w}$ / $\delta_{t}$ free for the parallel/series‐2CXM). When fixed parameters were held at their nominal (rather than GT) values, $k_{w}$ ARE increased to 29% (parallel‐2CXM), $\delta_{t}$ ARE increased to 5% (series‐2CXM). The ARE for $T_{\text{exch}}^{-1}$ was 33%.

#### 3.2.3 Simulation 3: Noisy simulation with fixed‐parameter error

This simulation was a practical identifiability analysis, with error in the fixed parameters (other than CBF and ATT) as well as including noise. The log–log relationship between noise (average GM signal variance, $\sigma^{2}$) and signal intensity, observed in a volunteer who underwent the 10‐repeat variant of the ME scan, was found to be: $\ln\left(\sigma^{2}\right)=1.3\ln\bar{\left(\Delta M\right)}-3.4$ (*R* ^2^ = 0.57).

This simulation was repeated for the locally structurally identifiable version of the model from Simulation 1. The mean and median fitted values for each parameter (which can be compared to the nominal values in Table 2) are provided, along with the median ARE. Both models had upwardly biased mean exchange rates ($k_{w}$ = 926 min^−1^ and $T_{\text{exch}}^{-1}=$3.6 × 10^10^ min^−1^ compared to the true mean of 250 min^−1^), but the medians ($k_{w}$ = 253 min^−1^ and $T_{\text{exch}}^{-1}=$225 min^−1^) were close to the true means. The AREs for each exchange time were similar to Simulation 2 (32% for $k_{w}$ and 33% for $T_{\text{exch}}^{-1}$).

### 3.3 Two‐compartment model fit to real data

Fitted parameter values extracted from the 25 participants are provided in Table 4. Two participants with outlying CBF values (by Tukey's criterion) were excluded. Initially, this fit was performed with both $T1_{t}$ and $k_{w}$ (or $\delta_{t}$) free, informed by the simulation results (Section 3.2). However, this resulted in 22 (of 23) participants' $k_{w}$ and $T1_{t}$ values hitting the lower bounds when using the parallel‐2CXM, and $\delta_{t}$ landing on its initial value in 21 participants when using the series‐2CXM. This may suggest non‐identifiability for both models when applied to the real data. To improve the identifiability of $k_{w}$ (and $\delta_{t}$), both models were re‐fitted with $T1_{t}$ fixed at 1.33 s. An additional simulation performed with all parameters but $k_{w}$ (or $\delta_{t}$) fixed (Table 3, Iteration 2*) showed that $k_{w}$ and $\delta_{t}$ can be expected to have AREs of 36% and 32% when fitted in this way.

**TABLE 4:** Results (fitted parameter values) from fitting each model to real data.

Model	$T2_{b}$ value	Parameter	Median (LQ, UQ)
Single‐compartment	N/A	CBF (ml min^−1^ 100 g^−1^)	68.3 (59.1, 78.0)
		ATT (s)	1.37 (1.27, 1.43)
Mono‐exponential		$T2_{t}$ (ms)	77.3 (74.6, 79.8)
		$T2_{t}$‐fit ARR (%)	4.34 × 10^−4^ (3.20 × 10^−4^, 7.23 × 10^−4^)
Parallel‐2CXM	110 ms	$k_{w}$ (min^−1^)	2.69 × 10^−3^ (2.62 × 10^−6^, 0.424)
		$k_{w}$‐fit ARR (%)	12.4 (10.5 15.7)
	80 ms	$k_{w}$ (min^−1^)	0.0190 (1.40 × 10^−3^, 0.188)
		$k_{w}$‐fit ARR (%)	28.5 (26.2, 32.5)
	165 ms	$k_{w}$ (min^−1^)	30.5 (12.2, 43.0)
		$k_{w}$‐fit ARR (%)	8.98 (7.79, 11.2)
Series‐2CXM	110 ms	$\delta_{t}$ (s)	2.00 (2.00 2.01)
		$T_{\text{exch}}^{-1}$ (min^−1^)	96.4 (81.4, 104)
		$\delta_{t}$‐fit ARR (%)	19.1 (16.5, 23.1)
	80 ms	$\delta_{t}$ (s)	2.00 (2.00, 2.01)
		$T_{\text{exch}}^{-1}$ (min^−1^)	96.4 (80.6, 104)
		$\delta_{t}$‐fit ARR (%)	30.7 (27.7, 33.4)
	165 ms	$\delta_{t}$ (s)	2.00 (2.00, 2.00)
		$T_{\text{exch}}^{-1}$ (min^−1^)	97.3 (82.4105)
		$\delta_{t}$‐fit ARR (%)	18.2 (16.6, 19.3)

*Note*: ARR is the mean absolute relative fit residual between the model prediction and the data. Results are shown for the nominal literature‐based fixed value of $T2_{b}$ (110 ms) as well as two other fixed values (80 ms, 165 ms) that were tested to assess the effect of changin*g* $T2_{b}$ on $k_{w}$.

**FIGURE 6:**
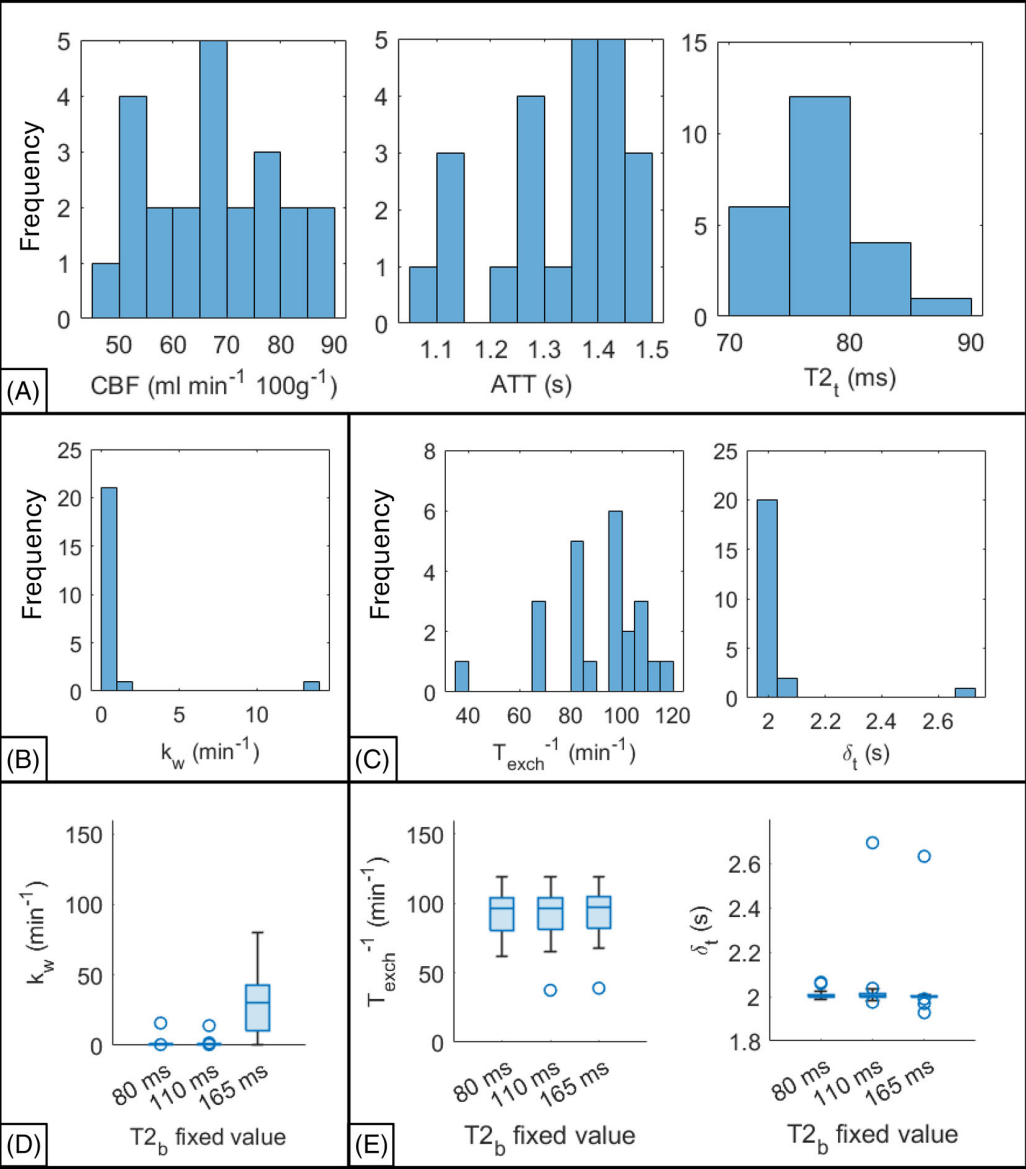
Distributions of fitted parameters for 25 cognitively normal older participants. (A) Results from the first two stages of the fit for CBF, ATT and *T*2_t_. (B) Results from the third stage of the fit, k_w_ with parallel‐2CXM). (C) $T_{\text{exch}}^{-1}$ and $\delta_{t}$ with the series‐2CXM. The bottom row shows the effect of changing the fixed values for $T2_{b}$ on $k_{w}$ with the parallel‐2CXM. (D) The effect on $T_{\text{exch}}^{-1}$ and $\delta_{t}$ using the series‐2CXM, (E). Outliers are plotted as circles. An outlier on panel D is not shown ($T2_{b}=$80 ms, $k_{w}=$885 min^−1^).

With this fit, both models yielded slower water exchange rates than the literature average. Median $k_{w}$ (parallel‐2CXM) was almost zero, whereas the series‐2CXM predicted exchange rates of 96.4 min^−1^. The parallel‐2CXM fit the data best with median absolute relative residuals (ARR) of 12.4% (vs. 19.1% for the series‐2CXM). Figure 6 (A–C) shows the fitted parameter distributions—despite being the only fitted parameters in the final fitting step, $k_{w}$ and $\delta_{t}$ mostly hit their lower bound (zero) and initial value (2.0 s), respectively.

Investigating the effect of changing the fixed $T2_{b}$ value on $k_{w}$ and $T_{\text{exch}}^{-1}$, for the parallel‐2CXM, $k_{w}$ increased when $T2_{b}$ increased to 165 ms (Figure 6D) however the series‐2CXM was relatively insensitive to this (Figure 6E).

## 4 DISCUSSION

Here a method for assessing model identifiability was presented and applied to BBB‐ASL water exchange models. Using simulations, we found that practically identifiable versions of the parallel‐ and series‐2CXMs could be attained by fixing all parameters except $k_{w}$ (or $\delta_{t}$) and $R1_{t}$. However, introducing a realistic degree of error into the fixed parameters resulted in low precision water exchange estimates. Model fits to data from 25 older participants yielded unexpectedly low $k_{w}$ (or $T_{\text{exch}}^{-1})$ estimates, and demonstrated bound‐ and initial‐value‐hitting behavior. These results highlight issues in model fitting which will be discussed.

This work demonstrated the utility of the sensitivity matrix approach for identifiability analysis, however, the result is specific to a single point in parameter space. The noise‐free (and fixed‐parameter‐error‐free) Monte‐Carlo simulation addressed this limitation by drawing samples from a larger region of physiologically plausible parameter values. The simulation showed additional parameters needed to be fixed in both 2CXM versions to achieve structural identifiability over a larger region of parameter space, with the final locally structurally identifiable version of both 2CXMs having only $R1_{t}$ and $k_{w}$ free.

The second Monte‐Carlo simulation introduced error into fixed parameters $R1_{b}$ and $R2_{b}$. We found large median water exchange AREs for both 2CXMs using this approach (29% for $k_{w}$ and 33% for $T_{\text{exch}}^{-1}$). Introducing noise in the third simulation increased the ARE for $k_{w}$ only slightly, and there was no increase to the ARE of $T_{\text{exch}}^{-1}$. This suggests that, if improvements to the scan were to be made, then obtaining accurate, individual‐specific estimates for any fixed parameters may be more useful than improving SNR (e.g., by taking more repeats). For example, individual‐specific $R1_{b}$ and $R2_{b}$ could be estimated by measuring hematocrit near the time of scanning and making assumptions about blood oxygenation and the degree of erythrocyte skimming in microvessels.^9^, ^49^, ^50^ Additionally, $R1_{t}$ could be estimated from other MRI scans.^51^ Such improvements are likely necessary if the ME‐ASL methods used here are to achieve clinical utility.

Although the practical identifiability analysis showed large AREs for $k_{w}$ and $T_{\text{exch}}^{-1}$, their median values approximated the true median of the uniform distribution from which the GT values were drawn. However, when both 2CXMs were fitted to real data, the fitted parameters ($k_{w}$ or $\delta_{t}$, and $R1_{t}$) tended to hit either the lower fitting bound ($k_{w}$ and $R1_{t}$) or the initial value ($\delta_{t}$). Fixing $R1_{t}$ did not resolve this. The series‐2CXM predicted $\delta_{t}$ = 2.0 s (the initial point) for almost all participants; this could be due to objective function flatness at the initial point (insensitivity to $\delta_{t}$) when applied to the real data. For the parallel‐2CXM, the tendency of $k_{w}$ to hit the lower bound (zero) could reflect a physiological reality (no BBB water permeability), but this would contradict other BBB water permeability studies to‐date. Performing practical identifiability by the profile‐likelihood method may shed light on the behavior of the objective function when fitting to real data.^26^, ^52^ This would be a good next step for future work, along with analyzing the identifiability of recently‐developed three‐compartment exchange models.^4^, ^6^ Optimizing the acquisition timing for $k_{w}$ or $\delta_{t}$ sensitivity using simulations^48^ could improve their precision but the utility hinges on the validity of the generative model. Overall, the contrast between the simulated and real fitting results indicates that further model validation work is needed to investigate possible model mis‐specification.

Identifiability analysis is only one step in the iterative model development process.^53^ Gaps remain in our understanding of BBB water transport,^54^ and a better understanding would help inform the structure of BBB‐ASL models. Insights in this area may be gained through multi‐disciplinary efforts, e.g., through other imaging modalities, histology and in‐vitro modeling. The use of BBB‐ASL in conjunction with orthogonal water permeability measurements, such as by H_2_^15^O PET, could serve as a useful validation experiment and has not yet been performed to the best of our knowledge.^55^

Although the work presented here cannot be used to validate one model or another, we observe that the two 2CXMs had different average residuals when fitted to real data. At the nominal $T2_{b}$ value of 110 ms, the parallel‐2CXM had lower median ARRs (12.4% vs. 19.1% for the series‐2CXM). The parallel‐2CXM also had lower ARRs compared to the series‐2CXM for the two alternative $T2_{b}$ values. The main conceptual differences between the two models are the inclusion of a time delay before labeled water can reach the EES in the series‐2CXM, and the inclusion of a water exchange term during the readout period in the parallel‐2CXM. Designing experiments to test these model features may be a good next step in model validation; smaller fitting residuals do not necessarily indicate that the parallel‐2CXM is a “better” model, since small residuals can also be achieved by a model that is a good signal representation but does not actually reflect the underlying physiology accurately.^53^

Fitting to real data demonstrated that, when using the parallel‐2CXM, the fitted value of $k_{w}$ was sensitive to the fixed value of $T2_{b}$. Although the sensitivity plots suggested that the sensitivity of the parallel‐2CXM to $T2_{b}$ was low (Figure 5A,B), Figure 6D shows that the fixed value of $k_{w}$ increased significantly at the higher value of $T2_{b}$ (165 ms), indicating that the sensitivity of the model output to $T2_{b}$ may increase at values >110 ms (at least when fitting to real data). Again, a profile‐likelihood analysis may provide more insight into the behavior of the objective function for different $T2_{b}$ values. Regardless of the underlying cause, this shows that the fixed value of $T2_{b}$ should be considered when comparing $k_{w}$ values across studies. Finally, fixing $T2_{b}$ at an assumed literature value may cause changes in blood oxygenation due to oxygen extraction down the capillary tree^56^ to be conflated with water exchange.^27^

Overall, the water exchange rates reported here are lower than those reported by other ASL studies.^32^ Even with $T2_{b}=$165 ms (the same fixed value as in other in‐human ME‐ASL BBB permeability studies,^4^, ^27^, ^28^, ^57^ which also utilize the parallel‐2CXM), the median $k_{w}$ value reported here for the parallel‐2CXM (31 min^−1^) was still much lower than previous ME‐ASL estimates,^4^, ^28^ including a previous report which included some of the same data (participants from “Cohort A” from^27^), but a different fitting method. This discrepancy could be due to differences in the image analysis and model fitting methods, such as the use of voxel‐wise fitting for $k_{w}$ estimation,^4^, ^27^, ^28^ and the use of individual‐specific estimates for $T2_{t}$ in this work. Whereas the $T_{\text{exch}}^{-1}$ values estimated by the series‐2CXM were more in‐line with previous literature estimates, this is likely an artifact of the fitting algorithm returning the initial guess for most participants—a reminder that physiologically plausible results are sometimes merely the outcome of researcher‐selected fitting bounds or initial values. It is also worth noting that ASL estimates in general disagree with previous estimates by H_2_^15^O PET^58^, ^59^; the reasons for the disagreement are still unclear and further work in this area may assist with BBB model validation.

There are limitations with this work that we would like to note. Firstly, the Monte Carlo simulations assumed that the fixed values for CBF, ATT, *and* $T2_{t}$ (which were able to be independently estimated from our data) were error‐free. In reality, these parameters would have error associated with them, likely resulting in larger $k_{w}$ ARE values. Second, only the effect of changing the fixed value of $T2_{b}$ on the value of $k_{w}$ was explored when fitting to real data—future analyses could assess how other commonly fixed parameters affect the fitted value of $k_{w}$, especially $T1_{b}$ which depends on hematocrit and blood oxygenation.^60^ Thirdly, the fidelity of the gray‐matter mask (derived from a T_1_‐weighted structural image) is limited by the relative coarseness of the ASL image it is applied to, and white‐matter and CSF partial‐volume effects may be a contributing factor to the poor fit quality observed. Finally, this work focused on ME‐ASL, in future the identifiability analysis methods used here could be applied to other BBB‐imaging techniques such as diffusion‐prepared‐ASL,^61^, ^62^ phase‐contrast‐ASL,^63^ and filter‐exchange imaging.^64^

## 5 CONCLUSIONS

Structural and practical identifiability analyses are useful tools to determine the accuracy and precision of model‐fitted parameters. In this work we performed identifiability analyses on variants of BBB‐ASL two‐compartment models. Simulations suggested a minimum water exchange error of 32% was achievable for our ME‐ASL protocol. However, the use of theoretically practically identifiable models with real data yielded unexpectedly small water exchange values, which may indicate the need for further model validation studies, and/or the use of profile‐likelihood analyses with real data. Future work could extend these analyses to different BBB‐ASL models and acquisition techniques and further model validation work may help to clarify our interpretation of ME‐ASL data and improve the accuracy and reliability of $k_{w}$ estimates. Ultimately our work shows that water exchange estimates depend on the model used and the values of fixed parameters, notably the T_2_ of blood.

## Supporting information


**Figure S1.** Identifiability signatures for the ME scan (parallel‐ 2CXM) (A: PLD = 1100 ms, B: PLD = 2100 ms). The singular values of S are shown in descending order, with a gap of more than three decades between singular values indicating non‐identifiability. The absolute values of the column vectors of $V^{T}$ show how the non‐identifiable parameters relate to the small singular value(s) of S. Only $\left|V^{T}\right|$ >0.001 values are shown. Sensitivity plots are shown in C (PLD = 1100 ms) and D (2100 ms).
**Figure S2.** Identifiability signatures for the ME scan (series‐2CXM) (A: PLD = 1100 ms, B: PLD = 2100 ms). The singular values of S are shown in descending order, with a gap of more than three decades between singular values indicating non‐identifiability. The absolute values of the column vectors of $V^{T}$ show how the non‐identifiable parameters relate to the small singular value(s) of S. Only $\left|V^{T}\right|$ >0.001 values are shown. Sensitivity plots are shown in C (PLD = 1100 ms) and D (2100 ms).
**Figure S3.** 1CM signal curve—magnetization in arbitrary units (AU) vs. time since the beginning of labelling, for the nominal physiological parameters given in Table 2. The analytical solution is overlaid (and obscured) by four numerical solutions, solved using the standard Heaviside AIF (Equation 2) (cyan), and the smooth approximate AIF (Equation 8) with c = 100 (red), 50 (green), and 25 s^−1^ (blue).

## Data Availability

The MATLAB code used for the identifiability analyses and fitting simulations, as well as image‐preprocessing code are available online at GitHub: https://github.com/tabithamanson/bbb_asl_identifiability.git (commit ccad7c6). De‐identified MRI data may be shared with other researchers solely for related research with the permission of co‐authors CM and/or LJT and the Dementia Prevention Research Clinics Management Committee.

## References

[1] Alsop DC , Detre JA , Golay X , et al. Recommended implementation of arterial spin‐labeled perfusion MRI for clinical applications: a consensus of the ISMRM perfusion study group and the European consortium for ASL in dementia. Magn Reson Med. 2015;73:102‐116. doi:10.1002/mrm.25197 24715426 PMC4190138

[2] St. Lawrence KS , Frank JA , McLaughlin AC . Effect of restricted water exchange on cerebral blood flow values calculated with arterial spin tagging: a theoretical investigation. Magn Reson Med. 2000;44:440‐449. doi:10.1002/1522-2594(200009)44:3<440::AID-MRM15>3.0.CO;2-6 10975897

[3] Alsop DC , Detre JA . Reduced transit‐time sensitivity in noninvasive magnetic resonance imaging of human cerebral blood flow. J Cereb Blood Flow Metab. 1996;16:1236‐1249. doi:10.1097/00004647-199611000-00019 8898697

[4] Mahroo A , Buck MA , Huber J , et al. Robust multi‐TE ASL‐based blood–brain barrier integrity measurements. Front Neurosci. 2021;15:15. doi:10.3389/fnins.2021.719676 PMC8678075 34924924

[5] Schidlowski M , Boland M , Rüber T , Stöcker T . Blood–brain barrier permeability measurement by biexponentially modeling whole‐brain arterial spin labeling data with multiple T_2_‐weightings. NMR Biomed. 2020;33:e4374. doi:10.1002/nbm.4374 32715563

[6] Shao X , Zhao C , Shou Q , St Lawrence KS , Wang DJJ . Quantification of blood–brain barrier water exchange and permeability with multidelay diffusion‐weighted pseudo‐continuous arterial spin labeling. Magn Reson Med. 2023;89:1990‐2004. doi:10.1002/mrm.29581 36622951 PMC10079266

[7] Wengler K , Bangiyev L , Canli T , Duong TQ , Schweitzer ME , He X . 3D MRI of whole‐brain water permeability with intrinsic diffusivity encoding of arterial labeled spin (IDEALS). Neuroimage. 2019;189:401‐414. doi:10.1016/j.neuroimage.2019.01.035 30682535

[8] Li W , van Zijl PCM . Quantitative theory for the transverse relaxation time of blood water. NMR Biomed. 2020;33:e4207. doi:10.1002/nbm.4207 32022362 PMC7322972

[9] Zhao JM , Clingman CS , Närväinen MJ , Kauppinen RA , van Zijl PCM . Oxygenation and hematocrit dependence of transverse relaxation rates of blood at 3T. Magn Reson Med. 2007;58:592‐597. doi:10.1002/mrm.21342 17763354

[10] An H , Lin W . Quantitative measurements of cerebral blood oxygen saturation using magnetic resonance imaging. J Cereb Blood Flow Metab. 2000;20:1225‐1236. doi:10.1097/00004647-200008000-00008 10950383 PMC4096835

[11] McHedlishvili G , Varazashvili M . Hematocrit in cerebral capillaries and veins under control and ischemic conditions. J Cereb Blood Flow Metab. 1987;7:739‐744. doi:10.1038/jcbfm.1987.128 3693429

[12] Miao H , Xia X , Perelson AS , Wu H . On identifiability of nonlinear ODE models and applications in viral dynamics. SIAM Rev Soc Ind Appl Math. 2011;53:3‐39. doi:10.1137/090757009 21785515 PMC3140286

[13] Joubert D , Stigter JD , Molenaar J . An efficient procedure to assist in the re‐parametrization of structurally unidentifiable models. Math Biosci. 2020;323:108328. doi:10.1016/j.mbs.2020.108328 32171772

[14] Buxton RB , Frank LR , Wong EC , Siewert B , Warach S , Edelman RR . A general kinetic model for quantitative perfusion imaging with arterial spin labeling. Magn Reson Med. 1998;40:383‐396. doi:10.1002/mrm.1910400308 9727941

[15] Parkes LM , Tofts PS . Improved accuracy of human cerebral blood perfusion measurements using arterial spin labeling: accounting for capillary water permeability. Magn Reson Med. 2002;48:27‐41. doi:10.1002/mrm.10180 12111929

[16] Tofts PS , Kermode AG . Measurement of the blood‐brain barrier permeability and leakage space using dynamic MR imaging. 1. Fundamental concepts. Magn Reson Med. 1991;17:357‐367. doi:10.1002/mrm.1910170208 2062210

[17] Wells JA , Siow B , Lythgoe MF , Thomas DL . Measuring biexponential transverse relaxation of the ASL signal at 9.4 T to estimate arterial oxygen saturation and the time of exchange of labeled blood water into cortical brain tissue. J Cereb Blood Flow Metab. 2013;33:215‐224. doi:10.1038/jcbfm.2012.156 23168531 PMC3564190

[18] Suzuki Y , Clement P , Dai W , et al. ASL lexicon and reporting recommendations: a consensus report from the ISMRM Open Science initiative for perfusion imaging (OSIPI). Magn Reson Med. 2024;91:1743‐1760. doi:10.1002/mrm.29815 37876299 PMC10950547

[19] Wang J , Alsop DC , Li L , et al. Comparison of quantitative perfusion imaging using arterial spin labeling at 1.5 and 4.0 tesla. Magn Reson Med. 2002;48:242‐254. doi:10.1002/mrm.10211 12210932

[20] Liu P , Uh J , Lu H . Determination of spin compartment in arterial spin labeling MRI. Magn Reson Med. 2010;65:120‐127. doi:10.1002/mrm.22601 PMC2994958 20740655

[21] Ohene Y , Harrison IF , Nahavandi P , et al. Non‐invasive MRI of brain clearance pathways using multiple echo time arterial spin labelling: an aquaporin‐4 study. Neuroimage. 2019;188:515‐523. doi:10.1016/j.neuroimage.2018.12.026 30557661 PMC6414399

[22] Chis OT , Banga JR , Balsa‐Canto E . Structural identifiability of systems biology models: a critical comparison of methods. PLoS One. 2011;6:e27755. doi:10.1371/journal.pone.0027755 22132135 PMC3222653

[23] Bellman R , Åström KJ . On structural identifiability. Math Biosci. 1970;7:329‐339. doi:10.1016/0025-5564(70)90132-X

[24] Wieland FG , Hauber AL , Rosenblatt M , Tönsing C , Timmer J . On structural and practical identifiability. Curr Opin Syst Biol. 2021;25:60‐69. doi:10.1016/j.coisb.2021.03.005

[25] Raue A , Kreutz C , Maiwald T , et al. Structural and practical identifiability analysis of partially observed dynamical models by exploiting the profile likelihood. Bioinformatics. 2009;25:1923‐1929. doi:10.1093/bioinformatics/btp358 19505944

[26] Conte M , Woodall RT , Gutova M , et al. Structural and practical identifiability of contrast transport models for DCE‐MRI. PLoS Comput Biol. 2024;20:e1012106. doi:10.1371/journal.pcbi.1012106 38748755 PMC11132485

[27] Morgan CA , Thomas DL , Shao X , et al. Measurement of blood–brain barrier water exchange rate using diffusion‐prepared and multi‐echo arterial spin labelling: comparison of quantitative values and age dependence. NMR Biomed. 2024;37:e5256. doi:10.1002/nbm.5256 39252500

[28] Mahroo A , Konstandin S , Günther M . Blood–brain barrier permeability to water measured using multiple echo time arterial spin labeling MRI in the aging human brain. J Magn Reson Imaging. 2023;59:1269‐1282. doi:10.1002/jmri.28874 37337979

[29] St. Lawrence K s , Owen D , Wang DJJ . A two‐stage approach for measuring vascular water exchange and arterial transit time by diffusion‐weighted perfusion MRI. Magn Reson Med. 2012;67:1275‐1284. doi:10.1002/mrm.23104 21858870 PMC4066380

[30] Günther M . Highly efficient accelerated acquisition of perfusion inflow series by cycled arterial spin labeling. Proceedings of the 16th Annual Meeting of ISMRM. International Society for Magnetic Resonance in Medicine; 2015:380.

[31] The MathWorks Inc . Solve nonstiff differential equations — variable order method – MATLAB ode113. The MathWorks Help Centre 2024 https://au.mathworks.com/help/matlab/ref/ode113.html Accessed March 14, 2024.

[32] Dickie BR , Parker GJM , Parkes LM . Measuring water exchange across the blood‐brain barrier using MRI. Prog Nucl Magn Reson Spectrosc. 2020;116:19‐39. doi:10.1016/j.pnmrs.2019.09.002 32130957

[33] The MathWorks Inc . Optimization Toolbox. 2022 https://www.mathworks.com

[34] The MathWorks Inc . Statistics and Machine Learning Toolbox. 2022 https://www.mathworks.com

[35] Juttukonda MR , Li B , Almaktoum R , et al. Characterizing cerebral hemodynamics across the adult lifespan with arterial spin labeling MRI data from the human connectome project‐aging. Neuroimage. 2021;230:117807. doi:10.1016/j.neuroimage.2021.117807 33524575 PMC8185881

[36] Zhang X , Petersen ET , Ghariq E , et al. In vivo blood T_1_ measurements at 1.5 T, 3 T, and 7 T. Magn Reson Med. 2013;70:1082‐1086. doi:10.1002/mrm.24550 23172845

[37] Wansapura JP , Holland SK , Dunn RS , Ball WS . NMR relaxation times in the human brain at 3.0 tesla. J Magn Reson Imaging. 1999;9:531‐538. doi:10.1002/(sici)1522-2586(199904)9:4<531::aid-jmri4>3.0.co;2-l 10232510

[38] Li W . Blood T_2_ and T_1_ Calculator. Blood T_2_, T_1_, Hct and Oxygenation Calculator. https://godzilla.kennedykrieger.org/cgi‐bin//bloodT2T1_cal.pl Accessed March 12, 2024.

[39] Cox EF , Gowland PA . Measuring T_2_ and T_2_' in the brain at 1.5T, 3T and 7T using a hybrid gradient echo‐spin echo sequence and EPI. Proceedings of the International Society for Magnetic Resonance in Medicine. Vol 16. ISMRM; 2008:1411.

[40] Leenders KL , Perani D , Lammertsma AA , et al. Cerebral blood flow, blood volume and oxygen utilization normal values and effect of age. Brain. 1990;113:27‐47. doi:10.1093/brain/113.1.27 2302536

[41] Tippett LJ , Cawston EE , Morgan CA , et al. Dementia prevention research clinic: a longitudinal study investigating factors influencing the development of Alzheimer's disease in Aotearoa, New Zealand. J R Soc N Z. 2023;53:489‐510. doi:10.1080/03036758.2022.2098780 39439970 PMC11459802

[42] Smith SM . Fast robust automated brain extraction. Hum Brain Mapp. 2002;17:143‐155. doi:10.1002/hbm.10062 12391568 PMC6871816

[43] Jenkinson M , Bannister P , Brady M , Smith S . Improved optimization for the robust and accurate linear registration and motion correction of brain images. Neuroimage. 2002;17:825‐841. doi:10.1016/s1053-8119(02)91132-8 12377157

[44] Smith SM , Jenkinson M , Woolrich MW , et al. Advances in functional and structural MR image analysis and implementation as FSL. Neuroimage. 2004;23:S208‐S219. doi:10.1016/j.neuroimage.2004.07.051 15501092

[45] Jenkinson M , Beckmann CF , Behrens TEJ , Woolrich MW , Smith SM . FMRIB software library (FSL). Neuroimage. 2012;62:782‐790. doi:10.1016/j.neuroimage.2011.09.015 21979382

[46] Chappell MA , Groves AR , Whitcher B , Woolrich MW . Variational Bayesian inference for a nonlinear forward model. IEEE Trans Signal Process. 2009;57:223‐236.

[47] Chappell MA , Groves AR , MacIntosh BJ , Donahue MJ , Jezzard P , Woolrich MW . Partial volume correction of multiple inversion time arterial spin labeling MRI data. Magn Reson Med. 2011;65:1173‐1183. doi:10.1002/mrm.22641 21337417

[48] Woods JG , Chappell MA , Okell TW . A general framework for optimizing arterial spin labeling MRI experiments. Magn Reson Med. 2019;81:2474‐2488. doi:10.1002/mrm.27580 30588656 PMC6492260

[49] Lu H , Clingman C , Golay X , van Zijl PCM . Determining the longitudinal relaxation time (T_1_) of blood at 3.0 tesla. Magn Reson Med. 2004;52:679‐682. doi:10.1002/mrm.20178 15334591

[50] Chen JJ , Pike BG . Human whole blood T_2_ relaxometry at 3 tesla. Magn Reson Med. 2009;61:249‐254. doi:10.1002/mrm.21858 19165880

[51] Stikov N , Boudreau M , Levesque IR , Tardif CL , Barral JK , Pike GB . On the accuracy of T_1_ mapping: searching for common ground. Magn Reson Med. 2015;73:514‐522. doi:10.1002/mrm.25135 24578189

[52] Wanika L , Egan JR , Swaminathan N , et al. Structural and practical identifiability analysis in bioengineering: a beginner's guide. J Biol Eng. 2024;18:20. doi:10.1186/s13036-024-00410-x 38438947 PMC11465550

[53] Novikov DS , Kiselev VG , Jespersen SN . On modeling. SIAM Rev. 2018;79:3172‐3193. doi:10.1002/mrm.27101 PMC5905348 29493816

[54] MacAulay N . Molecular mechanisms of brain water transport. Nat Rev Neurosci. 2021;22:326‐344. doi:10.1038/s41583-021-00454-8 33846637

[55] Moyaert P , Padrela BE , Morgan CA , et al. Imaging blood‐brain barrier dysfunction: a state‐of‐the‐art review from a clinical perspective. Front Aging Neurosci. 2023;15:15. doi:10.3389/fnagi.2023.1132077 PMC10150073 37139088

[56] Barrett MJP , Suresh V . Extra permeability is required to model dynamic oxygen measurements: evidence for functional recruitment? J Cereb Blood Flow Metab. 2013;33:1402‐1411. doi:10.1038/jcbfm.2013.74 23673433 PMC3764383

[57] Gregori J , Schuff N , Kern R , Günther M . T_2_‐based arterial spin labeling measurements of blood to tissue water transfer in human brain. J Magn Reson Imaging. 2013;37:332‐342. doi:10.1002/jmri.23822 23019041 PMC3554863

[58] Herscovitch P , Raichle ME , Kilbourn MR , Welch MJ . Positron emission tomographic measurement of cerebral blood flow and permeability – surface area product of water using [15O] water and [11C] butanol. J Cereb Blood Flow Metab. 1987;7:527‐542. doi:10.1038/jcbfm.1987.102 3498732

[59] Berridge MS , Adler LP , Nelson AD , et al. Measurement of human cerebral blood flow with [15O]butanol and positron emission tomography. J Cereb Blood Flow Metab. 1991;11:707‐715. doi:10.1038/jcbfm.1991.127 1874804

[60] Li W , Grgac K , Huang A , Yadav N , Qin Q , van Zijl PCM . Quantitative theory for the longitudinal relaxation time of blood water. Magn Reson Med. 2016;76:270‐281. doi:10.1002/mrm.25875 26285144 PMC4758918

[61] Wang J , Fernández‐Seara MA , Wang S , St. Lawrence KS . When perfusion meets diffusion: in vivo measurement of water permeability in human brain. J Cereb Blood Flow Metab. 2007;27:839‐849. doi:10.1038/sj.jcbfm.9600398 16969383

[62] Shao X , Ma SJ , Casey M , D'Orazio L , Ringman JM , Wang DJJ . Mapping water exchange across the blood–brain barrier using 3D diffusion‐prepared arterial spin labeled perfusion MRI. Magn Reson Med. 2019;81:3065‐3079. doi:10.1002/mrm.27632 30561821 PMC6414249

[63] Lin Z , Li Y , Su P , et al. Non‐contrast MR imaging of blood‐brain barrier permeability to water. Magn Reson Med. 2018;80:1507‐1520. doi:10.1002/mrm.27141 29498097 PMC6097906

[64] Powell E , Ohene Y , Battiston M , Dickie BR , Parkes LM , Parker GJM . Blood‐brain barrier water exchange measurements using FEXI: impact of modeling paradigm and relaxation time effects. Magn Reson Med. 2023;90:34‐50. doi:10.1002/mrm.29616 36892973 PMC10962589

