# Supplementary material for: On the structural and practical identifiability of multi‐echo BBB‐ASL tracer kinetic models
Source: Magn Reson Med. 2025 Sep 10;95(2):1205–22. doi: 10.1002/mrm.70075 (PMC12681319; doi:10.1002/mrm.70075)
Supplement: Supplementary file 1 — Figure S1. Identifiability signatures for the ME scan (parallel‐ 2CXM) (A: PLD = 1100 ms, B: PLD = 2100 ms). The singular values of S are shown in descending order, with a gap of more than three decades between singular values indicating non‐identifiability. The absolute values of the column vectors of VT show how the non‐identifiable parameters relate to the small singular value(s) of S. Only |VT| >0.001 values are shown. Sensitivity plots are shown in C (PLD = 1100 ms) and D (2100 ms). Figure S2. Identifiability signatures for the ME scan (series‐2CXM) (A: PLD = 1100 ms, B: PLD = 2100 ms). The singular values of S are shown in descending order, with a gap of more than three decades between singular values indicating non‐identifiability. The absolute values of the column vectors of VT show how the non‐identifiable parameters relate to the small singular value(s) of S. Only |VT| >0.001 values are shown. Sensitivity plots are shown in C (PLD = 1100 ms) and D (2100 ms). Figure S3. 1CM signal curve—magnetization in arbitrary units (AU) vs. time since the beginning of labelling, for the nominal physiological parameters given in Table 2. The analytical solution is overlaid (and obscured) by four numerical solutions, solved using the standard Heaviside AIF (Equation 2) (cyan), and the smooth approximate AIF (Equation 8) with c = 100 (red), 50 (green), and 25 s−1 (blue). [file MRM-95-1205-s001.docx]

Supplementary Material

Laplace transform structural identifiability analysis

In general, the set of differential equations describing a compartment model can be represented in terms of a vector of state variables, $\boldsymbol{x}\left( t \right)$; a matrix $\boldsymbol{A}\left( \boldsymbol{p} \right)$ containing the coefficients of the state variables in terms of the model parameters, $\boldsymbol{p}$; and the forcing term $\boldsymbol{B}\left( \boldsymbol{p} \right).\boldsymbol{u}\left( t \right)$, which describes the input to the system:

$$\frac{\partial\boldsymbol{x}}{\partial t}\mathbf{=}\boldsymbol{A}\left( \boldsymbol{p} \right)\mathbf{.}\boldsymbol{x}\left( t \right)+\boldsymbol{B}\left( \boldsymbol{p} \right)\mathbf{.}\boldsymbol{u}\left( t \right)$$

$$\boldsymbol{x}_{\mathbf{0}}=\boldsymbol{x}\left( t_{0}, \boldsymbol{p} \right)$$

[s-1]

where $\boldsymbol{x}_{\boldsymbol{0}}$ describes the state variables at the initial point $t=t_{0}.$^22^ Say $\boldsymbol{y}\left( t,\boldsymbol{p} \right)$, the model output, is some function of $\boldsymbol{x}\left( t \right)$; if $\boldsymbol{p}^{\boldsymbol{*}}$ is a set of parameters drawn from the same space as $\boldsymbol{p}$, then a model is globally structurally identifiable if $\boldsymbol{y}\left( t,\boldsymbol{p} \right)\neq\boldsymbol{y}\left( t,\boldsymbol{p}^{\boldsymbol{*}} \right)$ for all possible values of $\boldsymbol{p}^{\boldsymbol{*}}$ other than $\boldsymbol{p}^{\boldsymbol{*}}\boldsymbol{=p}$. A model can be locally (as opposed to globally) structurally identifiable if there is a neighborhood of $\boldsymbol{p}$ for which $\boldsymbol{y}\left( t,\boldsymbol{p} \right)\boldsymbol{=y}\left( t,\boldsymbol{p}^{\boldsymbol{*}} \right)$ implies $\boldsymbol{p}^{\boldsymbol{*}}\boldsymbol{=p}$.

To test this using the Laplace method, we can perform a Laplace transform of Eq. s-1 and rearrange to find the solution in the Laplace domain:

$$\boldsymbol{X}\left( s,\boldsymbol{p} \right)=\left( \boldsymbol{I}s-\boldsymbol{A} \right)^{-1}\boldsymbol{BU}\left( s \right)$$

$$\boldsymbol{Y}\left( s,\boldsymbol{p} \right)\mathcal{= L}\left\{ ƒ\left( \boldsymbol{x},\boldsymbol{p} \right) \right\}$$

[s-2]

We then let $\boldsymbol{Y}\left( s,\boldsymbol{p} \right)\equiv\boldsymbol{Y}\left( s,\boldsymbol{p}^{\boldsymbol{*}} \right)$ and equate coefficients of $s$ to analyse the structural identifiability. The 1CM (Eq. 1) can be re-expressed in the form of Eq. s-2 with $A= -{R1}_{t}, B=2{.M}_{0b}.\alpha{.e}^{-{R1}_{b}.ATT}.CBF$, and $u\left( t \right)=\theta\left( t-ATT \right)- \theta\left( t-(BL+ATT \right))$ and then transformed into the Laplace domain:

$$Y\left( s \right)=X\left( s \right)= \frac{2{.M}_{0b}.\alpha{.e}^{-{R1}_{b}.ATT}.CBF.e^{-ATT.s}\left( 1-e^{-BL.s} \right)}{s\left( s+{R1}_{t} \right)}$$

[s-3]

By defining $Y^{*}\left( s,p^{*} \right)\equiv Y\left( s,p \right)$ and cancelling known, assumed or independently measured variables ($M_{0b}, \alpha, BL)$from both sides, we obtain:

$$CBF.e^{-\left( {R1}_{b}+s \right)ATT}= {CBF}^{*}.e^{-\left( {{R1}_{b}}^{*}+s \right){ATT}^{*}}$$

[s-4]

$$s(s+{R1}_{t})={s(s+R1}_{t}^{*})$$

[s-5]

thus ${R1}_{t}$ is identifiable. For the remaining parameters, taking the natural log of both sides of Eq. s-4 and equate coefficients of $s$:

$$\ln\left( CBF \right)-\mathrm{ATT}\left( {R1}_{b}+s \right)=\ln\left( {CBF}^{*} \right)- \mathrm{ATT}^{*}\left( {R1}_{b}^{*}+s \right)$$

$$\therefore ATT= \mathrm{ATT}^{*}$$

$$\ln\left( CBF \right)-{ATT.R1}_{b}=\ln\left( {CBF}^{*} \right)- ATT. {R1}_{b}^{*}$$

therefore $ATT$ is also structurally identifiable, but one of either ${R1}_{b}$or $\mathrm{CBF}$ needs to be fixed in order to identify the other. This aligns with what is commonly performed in practice when using the 1CM to estimate CBF, where ${R1}_{b}$ is fixed at a value obtained from literature.^1^

ME Scan Identifiability Signatures


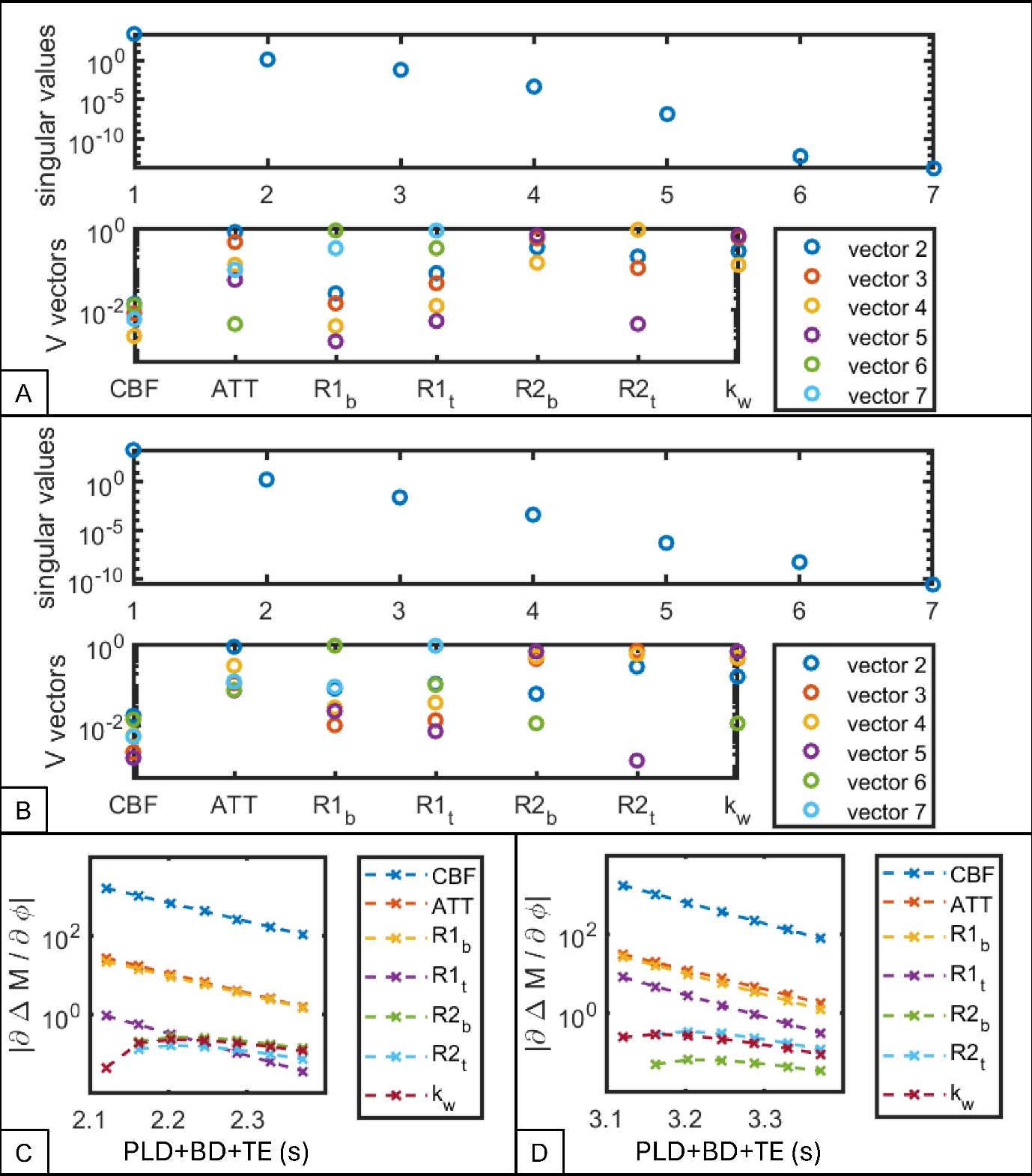


*Figure S1: Identifiability signatures for the ME scan (parallel- 2CXM) (A: PLD=1100 ms, B: PLD=2100 ms). The singular values of S are shown in descending order, with a gap of more than three decades between singular values indicating non-identifiability. The absolute values of the column vectors of* $\boldsymbol{V}^{T}$ *show how the non-identifiable parameters relate to the small singular value(s) of* $\boldsymbol{S}$***.*** *Only* $\boldsymbol{|V}^{T}|$ *> 0.001 values are shown. Sensitivity plots are shown in C (PLD=1100 ms) and D (2100 ms).*


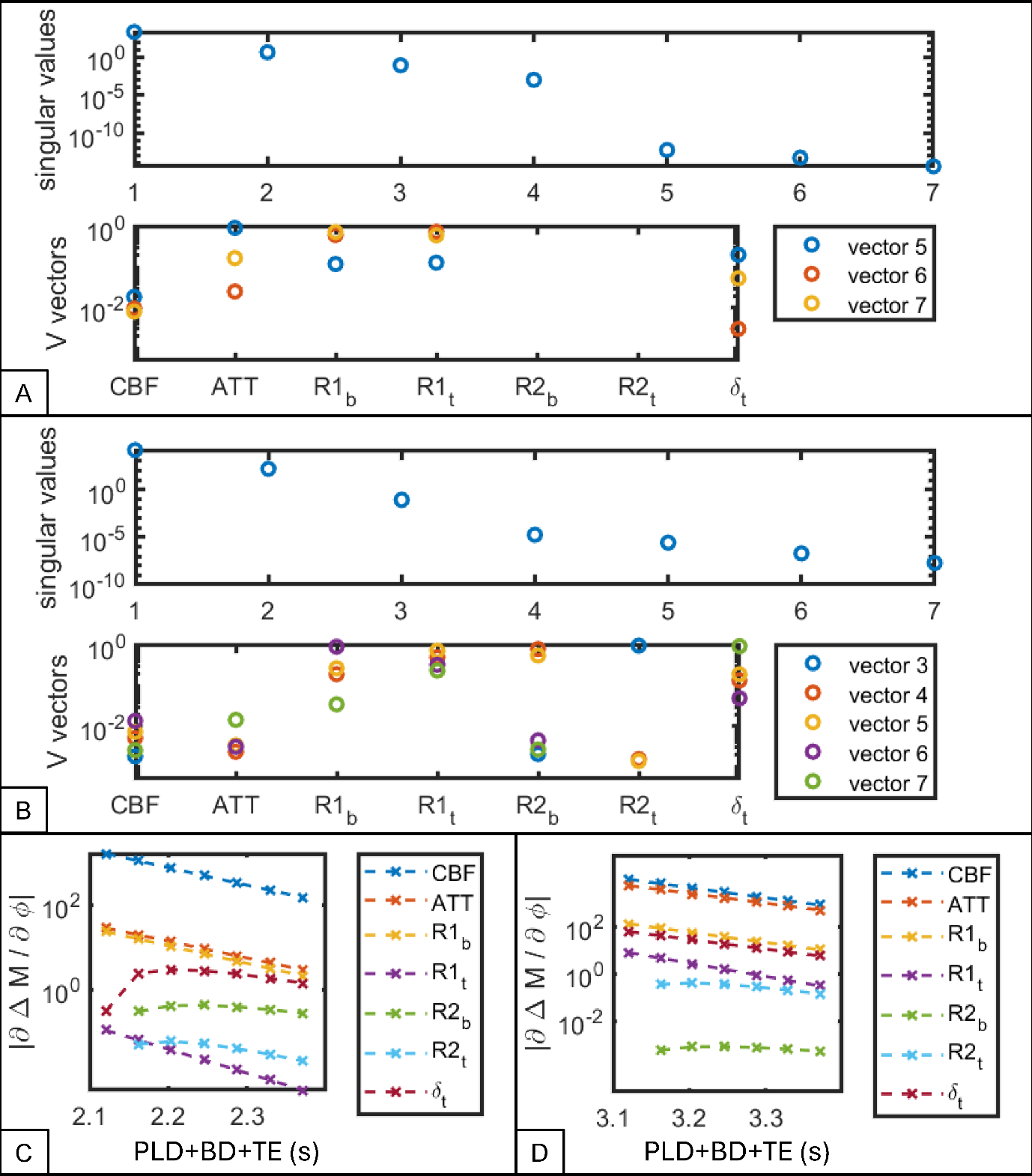


*Figure S2: Identifiability signatures for the ME scan (series-2CXM) (A: PLD=1100 ms, B: PLD=2100 ms). The singular values of* $\boldsymbol{S}$ *are shown in descending order, with a gap of more than three decades between singular values indicating non-identifiability. The absolute values of the column vectors of* $\boldsymbol{V}^{T}$ *show how the non-identifiable parameters relate to the small singular value(s) of* $\boldsymbol{S}$***.*** *Only* $\boldsymbol{|V}^{T}|$ *> 0.001 values are shown. Sensitivity plots are shown in C (PLD=1100 ms) and D (2100 ms).*

Single-compartment model solution with smooth AIF approximations


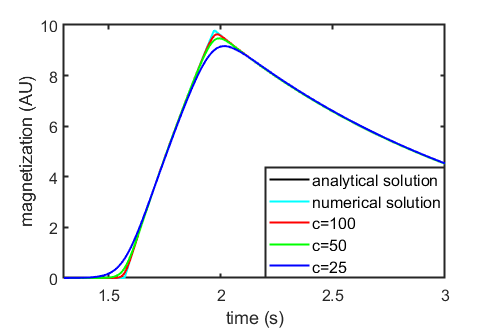


*Figure S3: 1CM signal curve – magnetization in arbitrary units (AU) vs. time since the beginning of labelling, for the nominal physiological parameters given in Table 2. The analytical solution is overlaid (and obscured) by four numerical solutions, solved using the standard Heaviside AIF (Eq. (2)) (cyan), and the smooth approximate AIF (Eq. (8)) with c=100 (red), 50 (green), and 25 s^-1^ (blue).*

The effect of changing the smoothness of the AIF approximation was assessed as follows. The rank of ***S*** was calculated for three successively smaller values of *c* (100, 50, 25 s^-1^) – see Eq. 8. As ***S*** was unchanged (rank=4), the largest value (*c*=100 s^-1^) was chosen as it most closely matched the analytical solution (Figure S3).
